# Supplementary material for: A mutation-induced drug resistance database (MdrDB)
Source: Commun Chem. 2023 Jun 14;6:123. doi: 10.1038/s42004-023-00920-7 (PMC10267113; doi:10.1038/s42004-023-00920-7)
Supplement: Supplementary file 2 — Supplementary Information [file 42004_2023_920_MOESM2_ESM.pdf]

Supplementary Material for A mutation-induced drug resistance database (MdrDB)

Ziyi Yang[1,†], Zhaofeng Ye[1,†], Jiezhong Qiu[1], Rongjun Feng[1], Danyu Li[1], Changyu Hsieh[1], Jonathan Allcock[1] and Shengyu Zhang[1,*]

[1]Tencent Quantum Laboratory, Shenzhen 518057, Guangdong, China.

[*]Corresponding author: shengyzhang@tencent.com.

[+]These authors contributed equally to this work.

DESCRIPTION: Some additional information about MdrDB is shown in the supplemental information.

This PDF file includes:

- Supplementary Figures 1-9
- Supplementary Tables 1-9
- Supplementary Note1: Model performance evaluation
- Supplementary Note2: Web design and interface

Contents

Supplementary Figures 3

Supplementary Tables 14

Supplementary Note1: Model performance evaluation 23

Scenario 1.1: Randomly split the samples (single substitution). 25

Scenario 1.2: 5-fold cross-validation (single substitution). 27

Scenario 1.3: Group 5-fold cross-validation (Uniprot ID). 28

Scenario 1.4: 5-fold nested cross-validation (protein sequence). 29

Scenario 1.5: Group 5-fold cross-validation (drug name). 31

Scenario 1.6: 5-fold nested cross-validation (SMILES). 32

Scenario 1.7: 25-fold nested cross-validation (amino acid type). 33

Scenario 1.8: 237-fold nested cross-validation (amino acid). 35

Scenario 2.1: Randomly split the samples (multiple substitutions). 36

Scenario 2.2: 5-fold cross-validation (multiple substitutions). 37

Scenario 3.1: Training on the single substitution, and test on the multiple substitutions. 39

Scenario 3.2: Training on the single substitution, test on the (deletion+indel+insertion+complex) mutation. 40

Scenario 4.1: Training on the single substitution, fine-tuning on the multiple substitutions (fine-tune : test = 8 : 2). 41

Scenario 4.2: Training on single substitution mutations, fine-tuning on (deletion+indel+insertion+complex) mutations (fine-tune : test = 8 : 2). 42

Supplementary Note2: Web design and interface 45

Browse. 45

Search. 45

Display. 45

Download of data, figures, and tables. 46

Supplementary References 48

Supplementary Figures

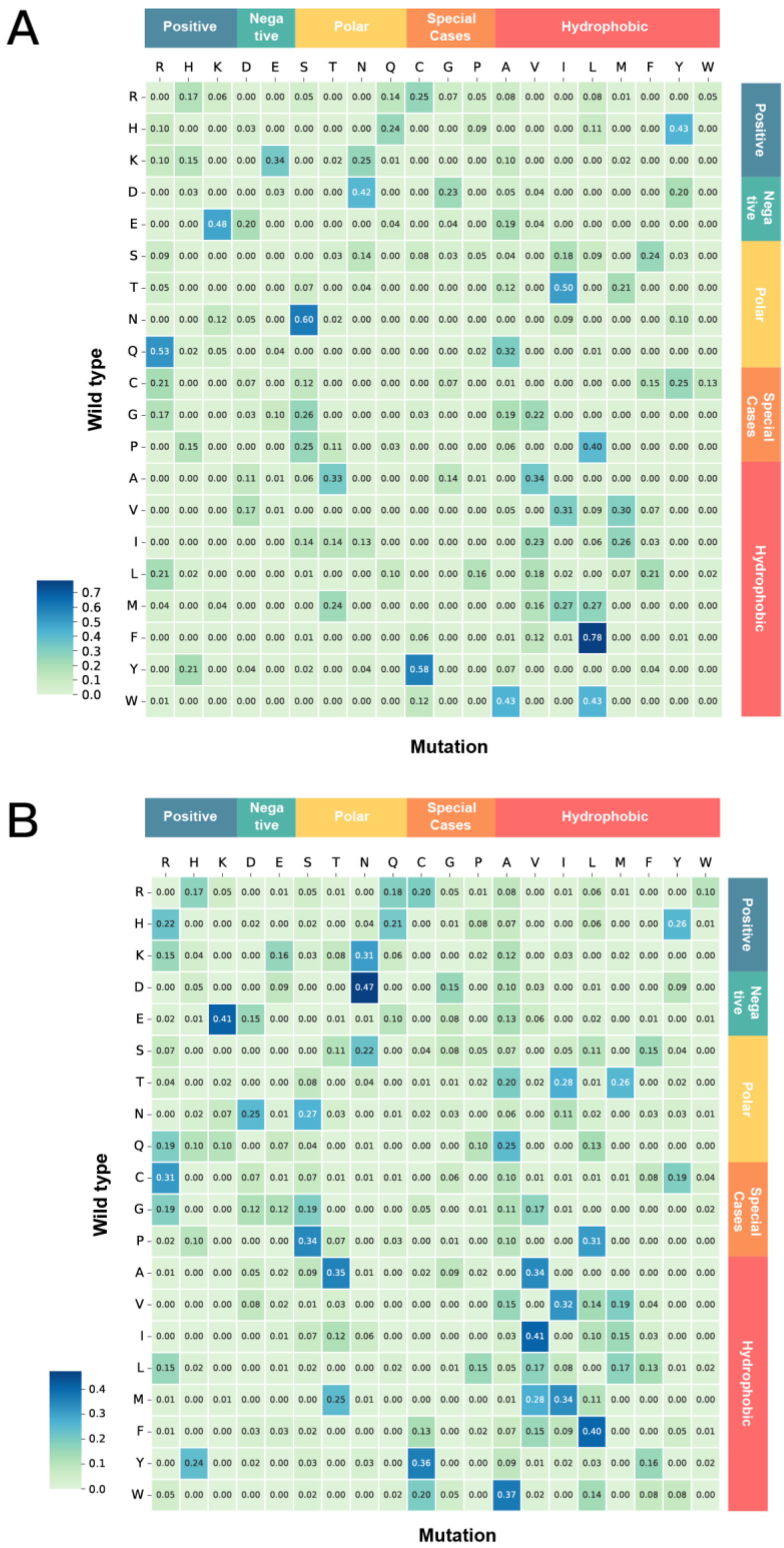

Supplementary Figure 1. Percentage of amino acid changes from substitution mutations in MdrDB. (A) MdrDB_FullSet and (B) MdrDB_CoreSet. The heatmap shows the percentage of amino acid changes from substitution mutations, using

different colors to show the percentage of changes in different amino acids. The number of samples for each amino acid in the wild type is displayed as a bar chart along the left axis of the plot; the number of samples for each amino acid in the mutation is displayed as a bar chart along the bottom axis of the plot. The 20 amino acids are divided into 5 categories: positive, negative, polar, hydrophobic, and special cases.

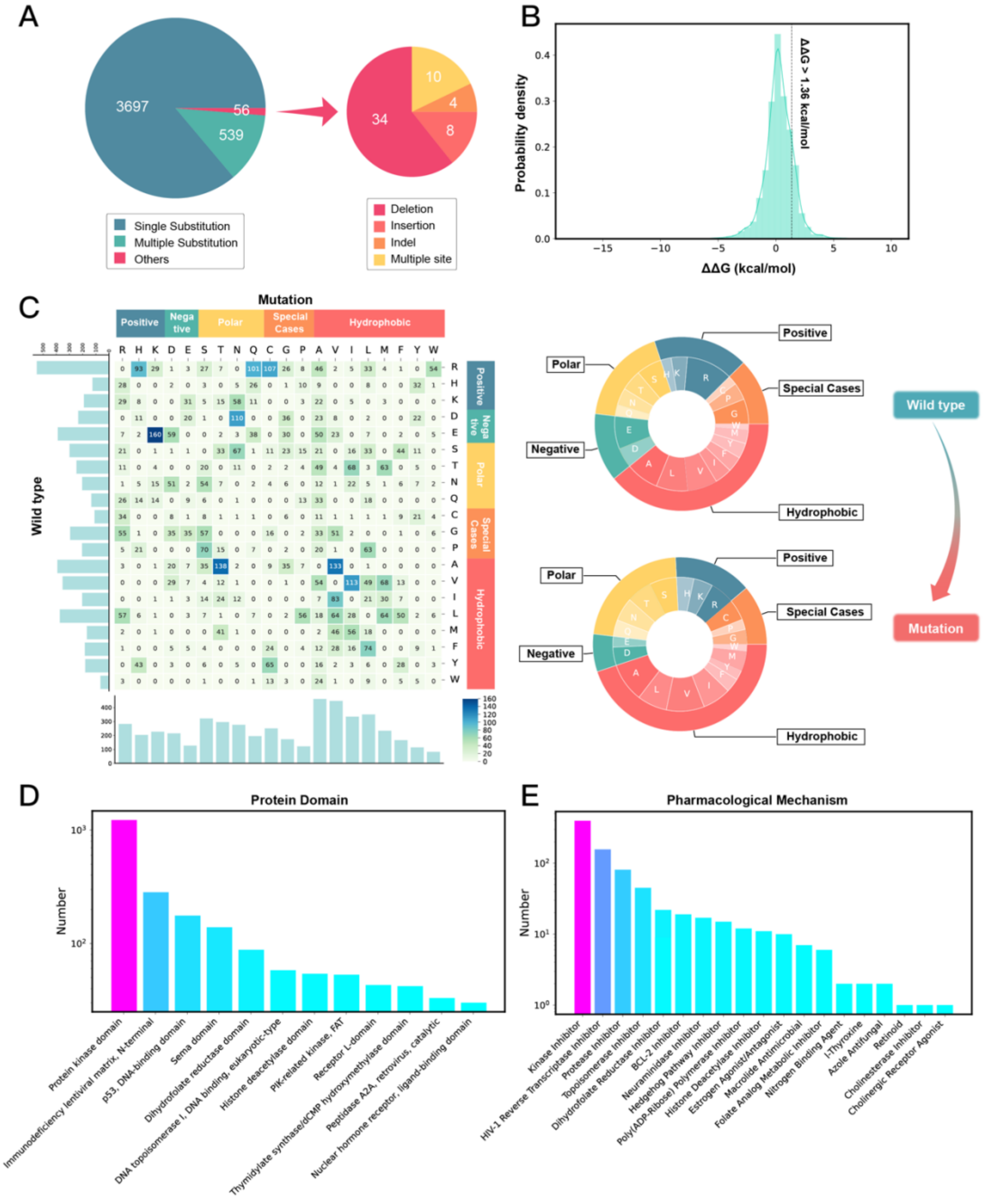

Supplementary Figure 2. Statistics of mutation information, △△G distribution, protein and drug annotations of MdrDB_CoreSet. (A) The number of samples in each mutation type. Non-repetitive "Uniprot-mutation-drug" is defined as a sample. Others include four mutation types: deletion, insertion, indel, and complex. (B) Histogram of the protein mutation-induced ligand binding affinity changes measured as △△G (kcal mol^{-1}). The line at △△G = 1.36 kcal mol^{-1} separates mutations defined as resistant from susceptible. (C) Number of amino acid changes from substitution mutations. (Left) The heatmap shows the number of amino acid changes from substitution mutations, using different colors to show the number of changes in different amino acids. The number of samples for each amino acid in the wild type is displayed as a bar chart along the left axis of the plot; the number of samples for each amino acid

in the mutation is displayed as a bar chart along the bottom axis of the plot. The 20 amino acids are divided into 5 categories: positive, negative, polar, hydrophobic, and special cases. (Right) Donut charts show the number of amino acids in the sample belonging to the substitution mutation. The proportion of each amino acid in wild-type samples is shown in the top, and the proportion of each amino acid in mutation is shown in the bottom. (D) Number of samples annotated into protein domains. The protein domain is represented on the x-axis, and the corresponding sample number is represented in log scale on the y-axis. (E) Number of samples annotated to pharmacological mechanisms. The pharmacological mechanisms are represented on the x-axis, and the corresponding sample number is represented in log scale on the y-axis.

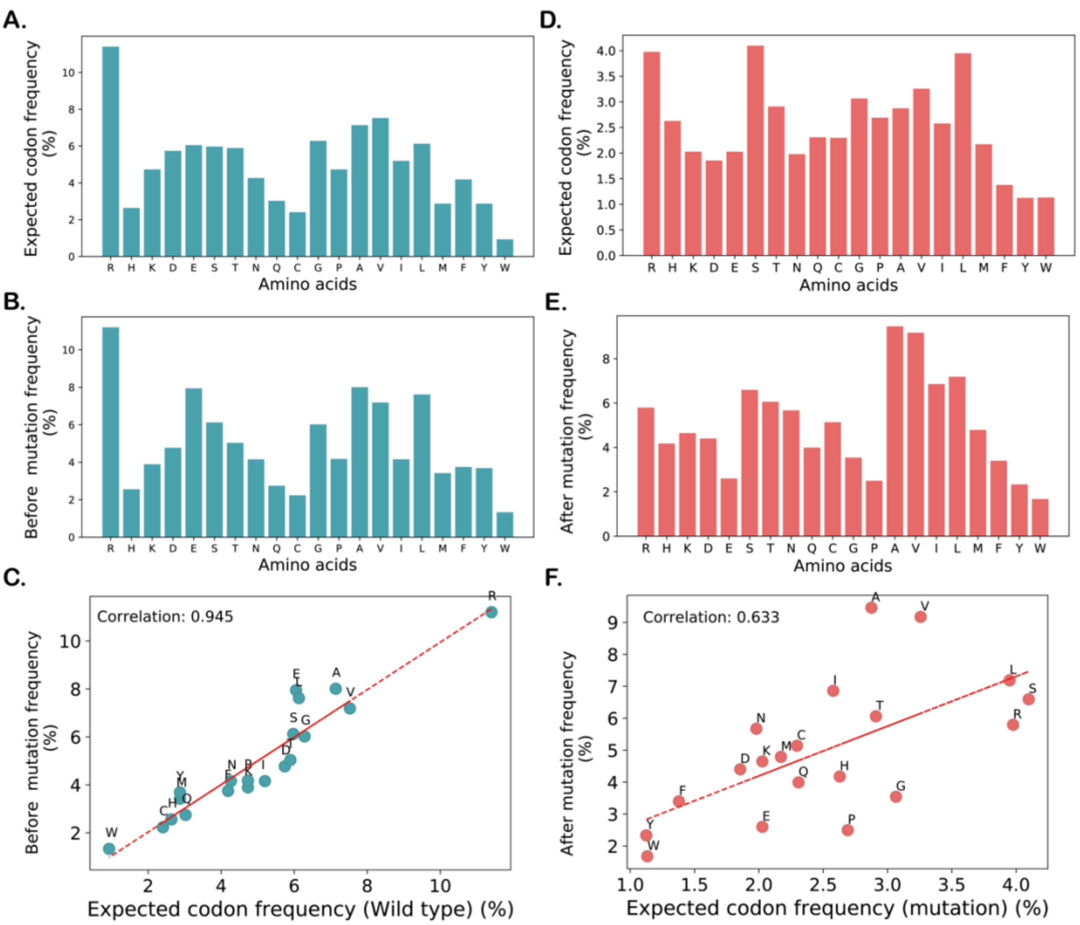

Supplementary Figure 3. Statistical analysis between the frequency of each amino acid type before and after mutation in MdrDB and the expected frequency calculated using codon frequencies from a previous study. (A) The expected codon frequency of each amino acid before mutation. (B) The frequency of each amino acid before mutation in MdrDB. (C) Correlation between the frequency of each amino acid before mutation in MdrDB and the expected frequency. (D) The expected codon frequency of each amino acid after mutation. (E) The frequency of each amino acid after mutation in MdrDB. (F) Correlation between the frequency of each amino acid after mutation in MdrDB and the expected frequency.

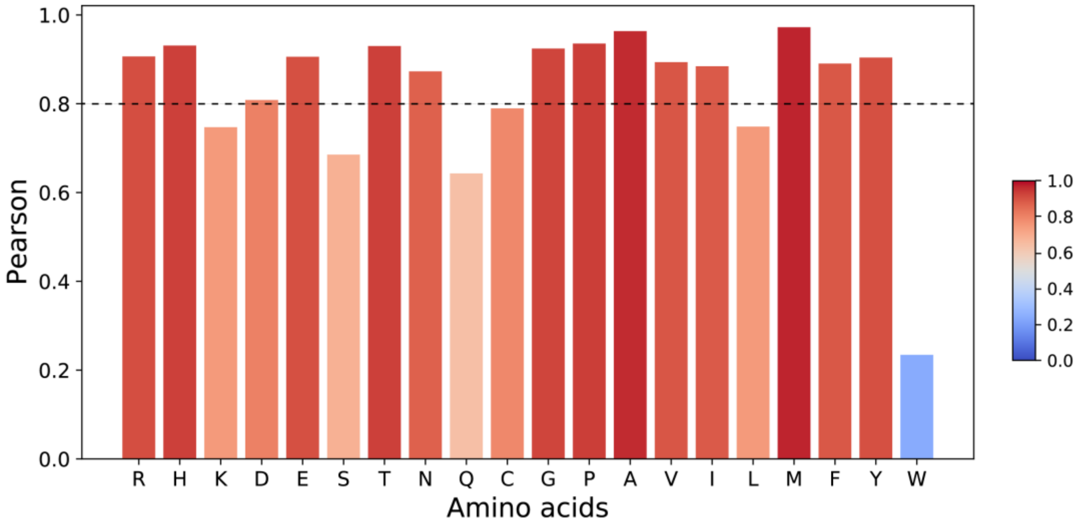

Supplementary Figure 4. Correlation between the mutation spectrum of each amino acid in the MdrDB single substitution subset and the mutation spectrum calculated based on codon frequency.

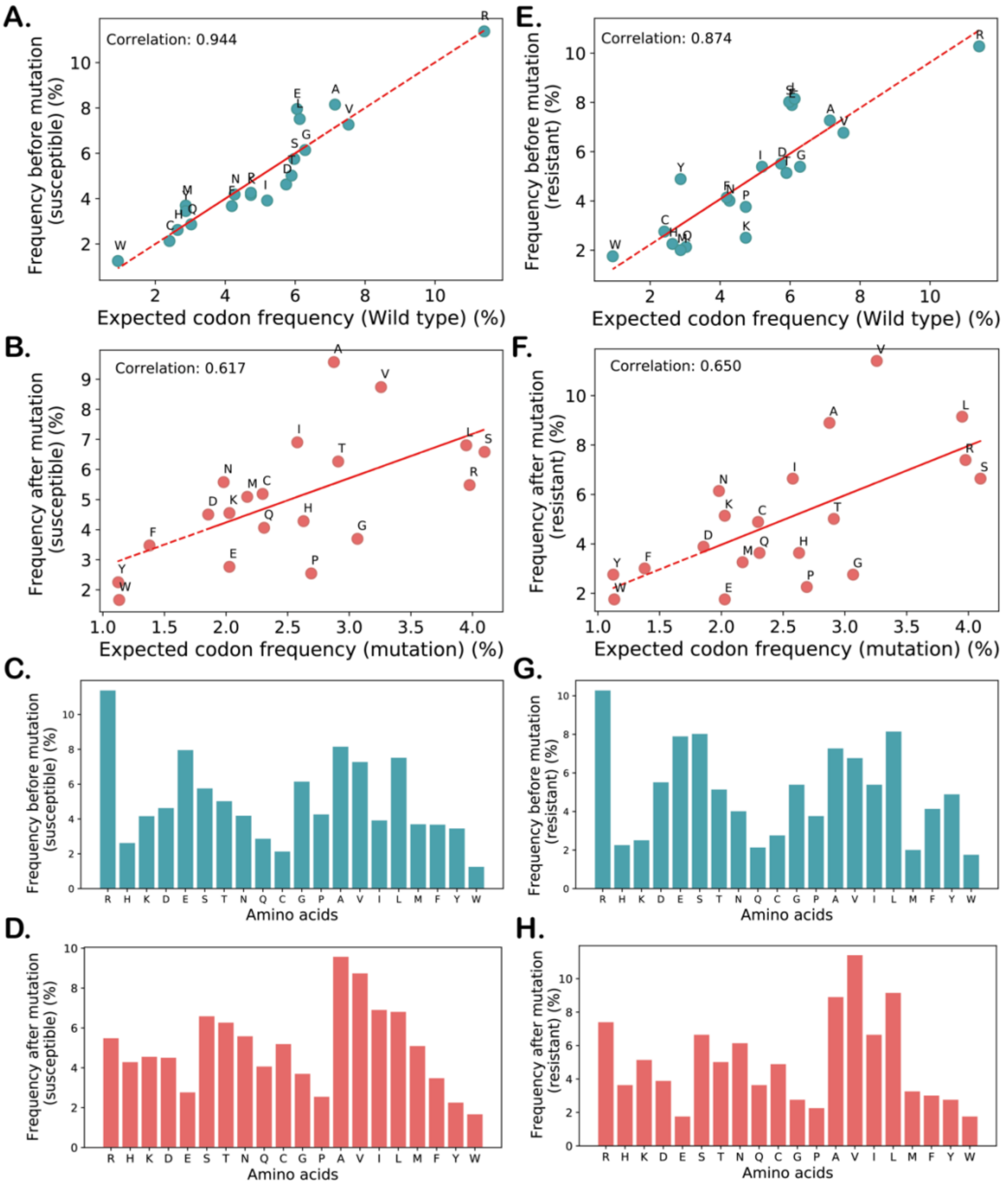

Supplementary Figure 5. Statistic information of each amino acid before and after mutation in the susceptible and resistant samples with the expected codon frequency. (A) Correlation between the frequency of each amino acid before mutation in the susceptible samples of MdrDB and the expected frequency. (B) Correlation between the frequency of each amino acid after mutation in the susceptible samples of MdrDB and the expected frequency. (C) The frequency of each amino acid type before mutation in the susceptible samples of MdrDB. (D) The frequency of each amino acid type after mutation in the susceptible samples of MdrDB. (E) Correlation between the frequency of each amino acid before mutation in the resistant samples of MdrDB and the expected frequency. (F) Correlation between the frequency of each amino acid after mutation in the resistant samples of MdrDB and the expected frequency. (G) The frequency of each amino acid type before mutation in the resistant samples of MdrDB. (H) The frequency of each amino acid type after mutation in the resistant samples of MdrDB.

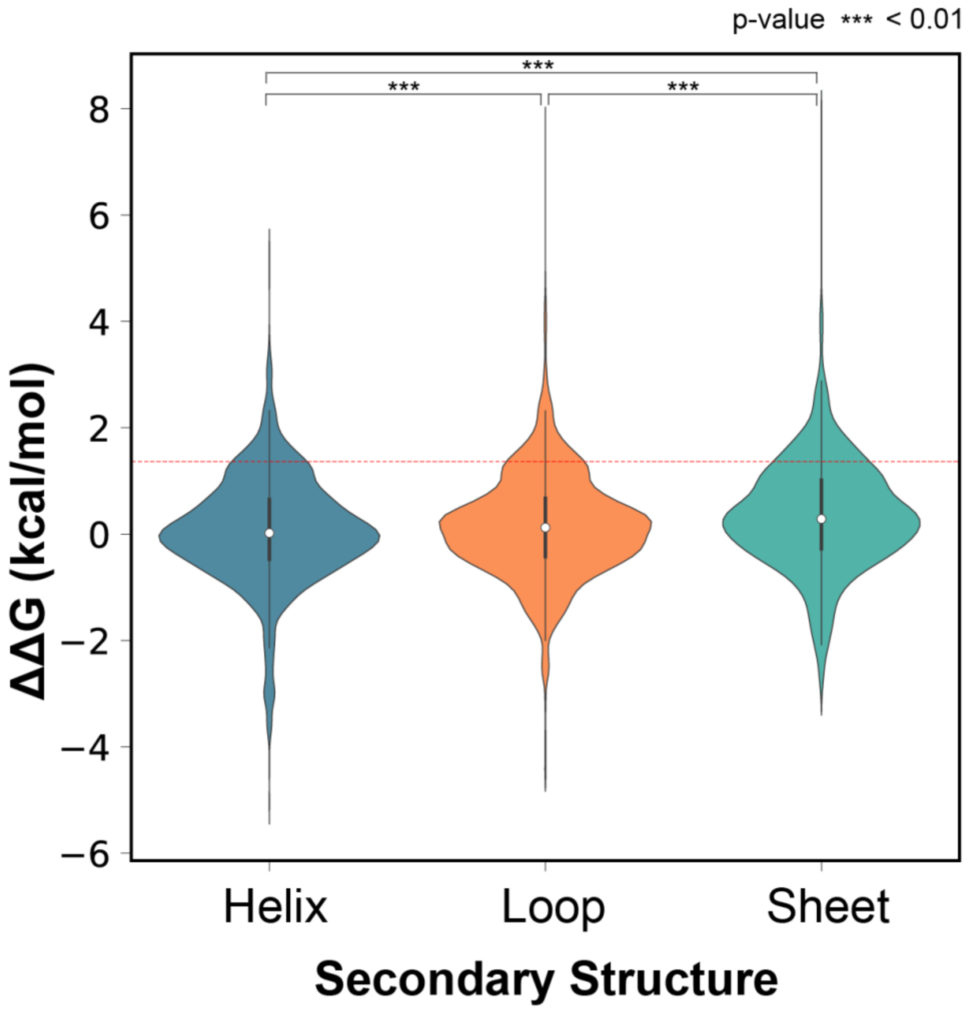

Supplementary Figure 6. Distribution of △△G values for the mutant site of amino acids with different predicted secondary structures: helix, loop, and sheet. 95% bootstrapped confidence intervals are shown.

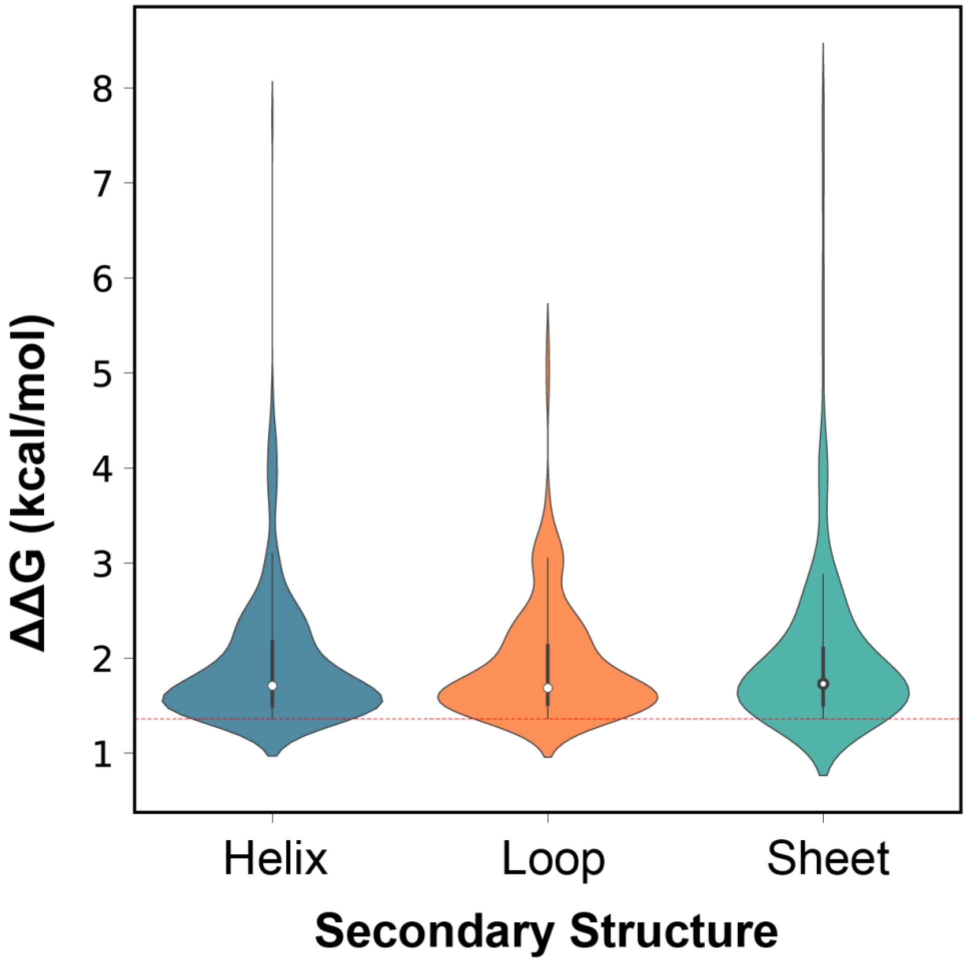

Supplementary Figure 7. Distribution of △△G values (>1.36 kcal mol^{-1}) for the mutant site of amino acids with different predicted secondary structures: helix, loop, and sheet. The x-axis represents the △△G values (kcal mol^{-1}), and the y-axis represents the protein's secondary structure.

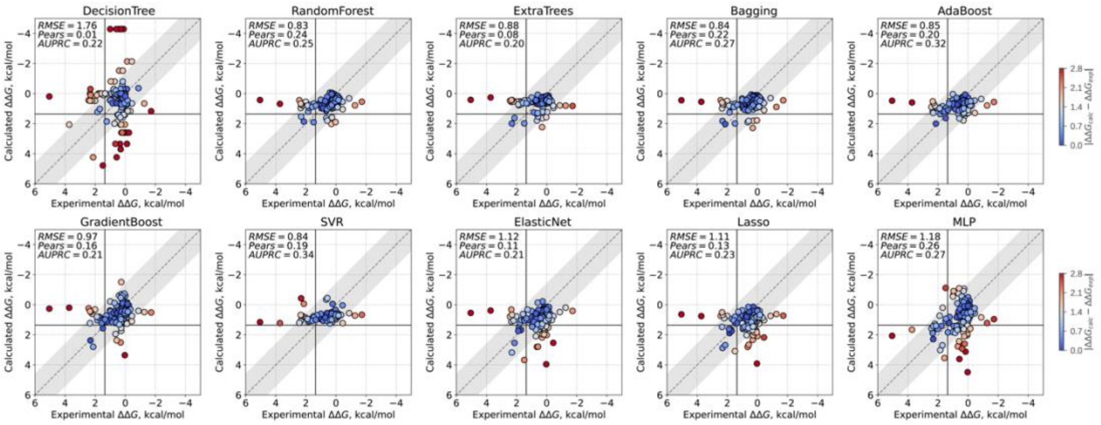

Supplementary Figure 8. Scatter plots of the experimental versus calculated ΔΔG values in Scenario 1. X-axis denotes the experimental ΔΔG values (kcal mol^{-1}). y-axis denotes the calculated ΔΔG value (kcal mol^{-1}). Each ΔΔG estimate is color-coded according to its absolute error w.r.t. the experimental ΔΔG value; at 300 K, the 1.4 kcal mol^{-1} error corresponds to a 10-fold error in the Kd change and 2.8 kcal mol^{-1} error corresponds to a 100-fold error in the Kd change.

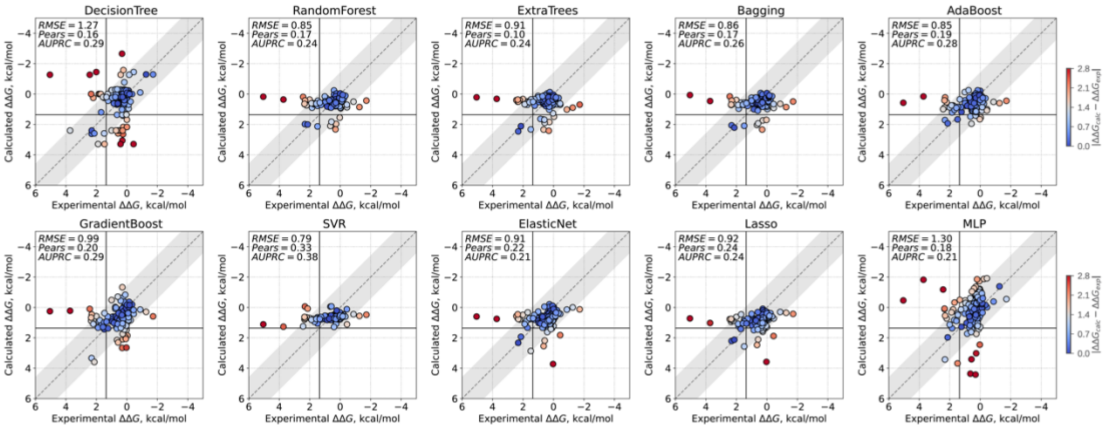

Supplementary Figure 9. Scatter plots of the experimental versus calculated ΔΔG values in Scenario 2. X-axis denotes the experimental ΔΔG values (kcal mol^{-1}). y-axis denotes the calculated ΔΔG value (kcal mol^{-1}). Each ΔΔG estimate is color-coded according to its absolute error w.r.t. the experimental ΔΔG value; at 300 K, the 1.4 kcal mol^{-1} error corresponds to a 10-fold error in the Kd change and 2.8 kcal mol^{-1} error corresponds to a 100-fold error in the Kd change.

Supplementary Tables

Supplementary Table 1. Overview of data sources in the MdrDB.

Data source		AIMMS	DepMap	GDSC	KinaseMD	Platinum	RET	TKI
Mutant structure source	# Processed	0	0	0	0	840	0	144
	# PyMOL mutated	5048	1347	86802	1334	0	456	0
	# AlphaFold2 folded	0	153	4413	0	0	0	0
Drug pose source	# Co-crystal	0	0	0	0	840	0	131
	# Docked	5048	1500	91215	1334	0	456	13
Mutation types	# Single substitution	224	1343	80190	992	689	456	144
	# Multiple substitution	4824	4	6656	342	151	0	0
	# Deletion	0	153	3075	0	0	0	0
	# Insertion	0	0	355	0	0	0	0
	# Indel	0	0	243	0	0	0	0
	# Complex	0	0	696	0	0	0	0

Supplementary Table 2. Number of amino acid changes from substitution mutations in MdrDB.

			Mutation Type																				
			Positive			Negative		Polar				Special Cases			Hydrophobic								
			R	H	K	D	E	S	T	N	Q	C	G	P	A	V	I	L	M	F	Y	W	COUNTS
Wild Type	Positive	R	0	2354	862	1	3	696	14	0	1903	3497	911	676	1082	2	13	1167	108	1	0	635	13925
		H	377	0	0	119	0	3	0	18	950	0	1	331	10	0	0	411	0	0	1663	1	3884
		K	458	653	0	0	1506	5	93	1127	54	0	0	3	467	0	17	0	69	0	0	0	4452
	Negative	D	0	147	0	0	131	1	0	1893	0	0	1064	0	224	166	0	2	0	0	903	0	4531
		E	7	2	3100	1270	0	0	2	3	268	0	255	0	1239	275	0	7	0	2	0	5	6435
	Polar	S	590	0	1	1	1	0	233	939	1	529	179	338	252	0	1243	624	0	1664	233	0	6828
		T	283	0	15	0	0	379	0	188	0	2	2	8	631	4	2682	3	1130	0	5	0	5332
		N	1	8	344	152	3	1777	50	0	3	5	7	0	14	2	272	5	1	6	294	2	2946
		Q	969	42	86	0	78	6	0	2	0	0	0	44	581	0	0	27	0	0	0	0	1835
	Special Cases	C	452	0	0	148	2	261	2	1	2	0	155	0	11	2	1	1	1	327	537	275	2178
		G	1780	1	0	331	1016	2673	0	0	0	300	0	2	1964	2337	2	0	0	1	0	37	10444
		P	12	685	0	0	0	1125	500	0	117	0	2	0	273	1	0	1835	0	0	0	0	4550
	Hydrophobic	A	3	0	1	859	58	450	2465	3	0	15	1057	45	0	2578	0	1	0	0	0	0	7535
		V	0	0	0	1548	86	4	12	1	0	0	2	0	426	0	2821	853	2744	604	0	0	9101
		I	0	0	3	1	3	607	621	572	0	0	0	0	7	989	0	263	1127	109	0	0	4302
		L	1416	146	1	0	3	43	2	0	668	0	2	1097	22	1192	116	0	464	1409	2	130	6713
		M	127	0	120	0	0	0	778	1	0	0	0	0	2	516	881	882	0	0	0	0	3307
		F	1	0	0	5	5	13	0	0	0	131	0	4	12	275	16	1738	0	0	21	1	2222
		Y	0	1157	0	229	0	93	0	231	0	3292	0	0	413	2	3	6	0	208	0	3	5637
		W	22	0	0	0	0	3	0	0	1	372	9	0	1297	2	0	1291	0	5	5	0	3007
		COUNTS	6498	5195	4533	4664	2895	8139	4772	4979	3967	8143	3646	2548	8927	8343	8067	9116	5644	4336	3663	1089	

Supplementary Table 3. Percentage of amino acid changes from substitution mutations in MdrDB.

			Mutation Type																			
			Positive			Negative		Polar			Special Cases			Hydrophobic								
			R	H	K	D	E	S	T	N	Q	C	G	P	A	V	I	L	M	F	Y	W
Wild Type	Positive	R	0.00%	16.90%	6.19%	0.01%	0.02%	5.00%	0.10%	0.00%	13.67%	25.11%	6.54%	4.85%	7.77%	0.01%	0.09%	8.38%	0.78%	0.01%	0.00%	4.56%
		H	9.71%	0.00%	0.00%	3.06%	0.00%	0.08%	0.00%	0.46%	24.46%	0.00%	0.03%	8.52%	0.26%	0.00%	0.00%	10.58%	0.00%	0.00%	42.82%	0.03%
		K	10.29%	14.67%	0.00%	0.00%	33.83%	0.11%	2.09%	25.31%	1.21%	0.00%	0.00%	0.07%	10.49%	0.00%	0.38%	0.00%	1.55%	0.00%	0.00%	0.00%
	Negative	D	0.00%	3.24%	0.00%	0.00%	2.89%	0.02%	0.00%	41.78%	0.00%	0.00%	23.48%	0.00%	4.94%	3.66%	0.00%	0.04%	0.00%	0.00%	19.93%	0.00%
		E	0.11%	0.03%	48.17%	19.74%	0.00%	0.00%	0.03%	0.05%	4.16%	0.00%	3.96%	0.00%	19.25%	4.27%	0.00%	0.11%	0.00%	0.03%	0.00%	0.08%
	Polar	S	8.64%	0.00%	0.01%	0.01%	0.01%	0.00%	3.41%	13.75%	0.01%	7.75%	2.62%	4.95%	3.69%	0.00%	18.20%	9.14%	0.00%	24.37%	3.41%	0.00%
		T	5.31%	0.00%	0.28%	0.00%	0.00%	7.11%	0.00%	3.53%	0.00%	0.04%	0.04%	0.15%	11.83%	0.08%	50.30%	0.06%	21.19%	0.00%	0.09%	0.00%
		N	0.03%	0.27%	11.68%	5.16%	0.10%	60.32%	1.70%	0.00%	0.10%	0.17%	0.24%	0.00%	0.48%	0.07%	9.23%	0.17%	0.03%	0.20%	9.98%	0.07%
		Q	52.81%	2.29%	4.69%	0.00%	4.25%	0.33%	0.00%	0.11%	0.00%	0.00%	0.00%	2.40%	31.66%	0.00%	0.00%	1.47%	0.00%	0.00%	0.00%	0.00%
	Special Cases	C	20.75%	0.00%	0.00%	6.80%	0.09%	11.98%	0.09%	0.05%	0.09%	0.00%	7.12%	0.00%	0.51%	0.09%	0.05%	0.05%	0.05%	15.01%	24.66%	12.63%
		G	17.04%	0.01%	0.00%	3.17%	9.73%	25.59%	0.00%	0.00%	0.00%	2.87%	0.00%	0.02%	18.81%	22.38%	0.02%	0.00%	0.00%	0.01%	0.00%	0.35%
		P	0.26%	15.05%	0.00%	0.00%	0.00%	24.73%	10.99%	0.00%	2.57%	0.00%	0.04%	0.00%	6.00%	0.02%	0.00%	40.33%	0.00%	0.00%	0.00%	0.00%
	Hydrophobic	A	0.04%	0.00%	0.01%	11.40%	0.77%	5.97%	32.71%	0.04%	0.00%	0.20%	14.03%	0.60%	0.00%	34.21%	0.00%	0.01%	0.00%	0.00%	0.00%	0.00%
		V	0.00%	0.00%	0.00%	17.01%	0.95%	0.04%	0.13%	0.01%	0.00%	0.00%	0.02%	0.00%	4.68%	0.00%	31.00%	9.37%	30.15%	6.64%	0.00%	0.00%
		I	0.00%	0.00%	0.07%	0.02%	0.07%	14.11%	14.44%	13.30%	0.00%	0.00%	0.00%	0.00%	0.16%	22.99%	0.00%	6.11%	26.20%	2.53%	0.00%	0.00%
		L	21.09%	2.17%	0.01%	0.00%	0.04%	0.64%	0.03%	0.00%	9.95%	0.00%	0.03%	16.34%	0.33%	17.76%	1.73%	0.00%	6.91%	20.99%	0.03%	1.94%
		M	3.84%	0.00%	3.63%	0.00%	0.00%	0.00%	23.53%	0.03%	0.00%	0.00%	0.00%	0.00%	0.06%	15.60%	26.64%	26.67%	0.00%	0.00%	0.00%	0.00%
		F	0.05%	0.00%	0.00%	0.23%	0.23%	0.59%	0.00%	0.00%	0.00%	5.90%	0.00%	0.18%	0.54%	12.38%	0.72%	78.22%	0.00%	0.00%	0.95%	0.05%
		Y	0.00%	20.53%	0.00%	4.06%	0.00%	1.65%	0.00%	4.10%	0.00%	58.40%	0.00%	0.00%	7.33%	0.04%	0.05%	0.11%	0.00%	3.69%	0.00%	0.05%
		W	0.73%	0.00%	0.00%	0.00%	0.00%	0.10%	0.00%	0.00%	0.03%	12.37%	0.30%	0.00%	43.13%	0.07%	0.00%	42.93%	0.00%	0.17%	0.17%	0.00%

Supplementary Table 4. Number of amino acid changes from substitution mutations in the coreset of MdrDB.

			Mutation Type																			
			Positive			Negative		Polar				Special Cases			Hydrophobic							
			R	H	K	D	E	S	T	N	Q	C	G	P	A	V	I	L	M	F	Y	W	COUNTS
Wild Type	Positive	R	0	93	29	1	3	27	7	0	101	107	26	8	46	2	5	33	4	1	0	54	547
		H	28	0	0	2	0	3	0	5	26	0	1	10	9	0	0	8	0	0	32	1	125
		K	29	8	0	0	31	5	15	58	11	0	0	3	22	0	5	0	3	0	0	0	190
	Negative	D	0	11	0	0	20	1	0	110	0	0	36	0	23	8	0	2	0	0	22	0	233
		E	7	2	160	59	0	0	2	3	38	0	30	0	50	23	0	7	0	2	0	5	388
	Polar	S	21	0	1	1	1	0	33	67	1	11	23	15	21	0	16	33	0	44	11	0	299
		T	11	0	4	0	0	20	0	11	0	2	2	4	49	4	68	3	63	0	5	0	246
		N	1	5	15	51	2	54	7	0	2	4	6	0	12	1	22	5	1	6	7	2	203
		Q	26	14	14	0	9	6	0	1	0	0	0	13	33	0	0	18	0	0	0	0	134
	Special Cases	C	34	0	0	8	1	8	1	1	1	0	6	0	11	1	1	1	1	9	21	4	109
		G	55	1	0	35	35	57	0	0	0	16	0	2	33	51	2	0	0	1	0	6	294
		P	5	21	0	0	0	70	15	0	7	0	2	0	20	1	0	63	0	0	0	0	204
	Hydrophobic	A	3	0	1	20	7	35	138	2	0	9	35	7	0	133	0	1	0	0	0	0	391
		V	0	0	0	29	7	4	12	1	0	0	1	0	54	0	113	49	68	13	0	0	351
		I	0	0	1	1	3	14	24	12	0	0	0	0	7	83	0	21	30	7	0	0	203
		L	57	6	1	0	3	7	1	0	7	0	2	56	18	64	28	0	64	50	2	6	372
		M	2	0	1	0	0	0	41	1	0	0	0	0	2	46	56	18	0	0	0	0	167
		F	1	0	0	5	5	4	0	0	0	24	0	4	12	28	16	74	0	0	9	1	183
		Y	0	43	0	3	0	6	0	5	0	65	0	0	16	2	3	6	0	28	0	3	180
		W	3	0	0	0	0	1	0	0	1	13	3	0	24	1	0	9	0	5	5	0	65
	COUNTS		283	204	227	215	127	322	296	277	195	251	173	122	462	448	335	351	234	166	114	82	

Supplementary Table 5. Percentage of amino acid changes from substitution mutations in the coreset of MdrDB.

			Mutation Type																			
			Positive			Negative		Polar			Special Cases			Hydrophobic								
			R	H	K	D	E	S	T	N	Q	C	G	P	A	V	I	L	M	F	Y	W
Wild Type	Positive	R	0.00%	17.00%	5.30%	0.18%	0.55%	4.94%	1.28%	0.00%	18.46%	19.56%	4.75%	1.46%	8.41%	0.37%	0.91%	6.03%	0.73%	0.18%	0.00%	9.87%
		H	22.40%	0.00%	0.00%	1.60%	0.00%	2.40%	0.00%	4.00%	20.80%	0.00%	0.80%	8.00%	7.20%	0.00%	0.00%	6.40%	0.00%	0.00%	25.60%	0.80%
		K	15.26%	4.21%	0.00%	0.00%	16.32%	2.63%	7.89%	30.53%	5.79%	0.00%	0.00%	1.58%	11.58%	0.00%	2.63%	0.00%	1.58%	0.00%	0.00%	0.00%
	Negative	D	0.00%	4.72%	0.00%	0.00%	8.58%	0.43%	0.00%	47.21%	0.00%	0.00%	15.45%	0.00%	9.87%	3.43%	0.00%	0.86%	0.00%	0.00%	9.44%	0.00%
		E	1.80%	0.52%	41.24%	15.21%	0.00%	0.00%	0.52%	0.77%	9.79%	0.00%	7.73%	0.00%	12.89%	5.93%	0.00%	1.80%	0.00%	0.52%	0.00%	1.29%
	Polar	S	7.02%	0.00%	0.33%	0.33%	0.33%	0.00%	11.04%	22.41%	0.33%	3.68%	7.69%	5.02%	7.02%	0.00%	5.35%	11.04%	0.00%	14.72%	3.68%	0.00%
		T	4.47%	0.00%	1.63%	0.00%	0.00%	8.13%	0.00%	4.47%	0.00%	0.81%	0.81%	1.63%	19.92%	1.63%	27.64%	1.22%	25.61%	0.00%	2.03%	0.00%
		N	0.49%	2.46%	7.39%	25.12%	0.99%	26.60%	3.45%	0.00%	0.99%	1.97%	2.96%	0.00%	5.91%	0.49%	10.84%	2.46%	0.49%	2.96%	3.45%	0.99%
		Q	19.40%	10.45%	10.45%	0.00%	6.72%	4.48%	0.00%	0.75%	0.00%	0.00%	0.00%	9.70%	24.63%	0.00%	0.00%	13.43%	0.00%	0.00%	0.00%	0.00%
	Special Cases	C	31.19%	0.00%	0.00%	7.34%	0.92%	7.34%	0.92%	0.92%	0.92%	0.00%	5.50%	0.00%	10.09%	0.92%	0.92%	0.92%	0.92%	8.26%	19.27%	3.67%
		G	18.71%	0.34%	0.00%	11.90%	11.90%	19.39%	0.00%	0.00%	0.00%	5.44%	0.00%	0.68%	11.22%	17.35%	0.68%	0.00%	0.00%	0.34%	0.00%	2.04%
		P	2.45%	10.29%	0.00%	0.00%	0.00%	34.31%	7.35%	0.00%	3.43%	0.00%	0.98%	0.00%	9.80%	0.49%	0.00%	30.88%	0.00%	0.00%	0.00%	0.00%
	Hydrophobic	A	0.77%	0.00%	0.26%	5.12%	1.79%	8.95%	35.29%	0.51%	0.00%	2.30%	8.95%	1.79%	0.00%	34.02%	0.00%	0.26%	0.00%	0.00%	0.00%	0.00%
		V	0.00%	0.00%	0.00%	8.26%	1.99%	1.14%	3.42%	0.28%	0.00%	0.00%	0.28%	0.00%	15.38%	0.00%	32.19%	13.96%	19.37%	3.70%	0.00%	0.00%
		I	0.00%	0.00%	0.49%	0.49%	1.48%	6.90%	11.82%	5.91%	0.00%	0.00%	0.00%	0.00%	3.45%	40.89%	0.00%	10.34%	14.78%	3.45%	0.00%	0.00%
		L	15.32%	1.61%	0.27%	0.00%	0.81%	1.88%	0.27%	0.00%	1.88%	0.00%	0.54%	15.05%	4.84%	17.20%	7.53%	0.00%	17.20%	13.44%	0.54%	1.61%
		M	1.20%	0.00%	0.60%	0.00%	0.00%	0.00%	24.55%	0.60%	0.00%	0.00%	0.00%	0.00%	1.20%	27.54%	33.53%	10.78%	0.00%	0.00%	0.00%	0.00%
		F	0.55%	0.00%	0.00%	2.73%	2.73%	2.19%	0.00%	0.00%	0.00%	13.11%	0.00%	2.19%	6.56%	15.30%	8.74%	40.44%	0.00%	0.00%	4.92%	0.55%
		Y	0.00%	23.89%	0.00%	1.67%	0.00%	3.33%	0.00%	2.78%	0.00%	36.11%	0.00%	0.00%	8.89%	1.11%	1.67%	3.33%	0.00%	15.56%	0.00%	1.67%
		W	4.62%	0.00%	0.00%	0.00%	0.00%	1.54%	0.00%	0.00%	1.54%	20.00%	4.62%	0.00%	36.92%	1.54%	0.00%	13.85%	0.00%	7.69%	7.69%	0.00%

Supplementary Table 6. Percentage of amino acid changes from substitution mutations calculated from codon frequency [1].

	R	H	K	D	E	S	T	N	Q	C	G	P	A	V	I	L	M	F	Y	W
R	0.00	18.21	3.71	0.00	0.00	5.43	1.14	0.00	16.71	18.21	10.64	6.64	0.00	0.00	0.29	5.14	0.57	0.00	0.00	13.29
H	23.75	0.00	0.00	10.00	0.00	0.00	0.00	8.75	21.25	0.00	0.00	7.50	0.00	0.00	0.00	7.50	0.00	0.00	21.25	0.00
K	31.67	0.00	0.00	0.00	25.50	0.00	7.33	16.83	11.33	0.00	0.00	0.00	0.00	0.00	2.00	0.00	5.33	0.00	0.00	0.00
D	0.00	11.71	0.00	0.00	18.87	0.00	0.00	24.52	0.00	0.00	25.62	0.00	8.26	4.41	0.00	0.00	0.00	0.00	6.61	0.00
E	0.00	0.00	31.47	16.78	0.00	0.00	0.00	0.00	14.69	0.00	20.94	0.00	8.84	7.28	0.00	0.00	0.00	0.00	0.00	0.00
S	11.52	0.00	0.00	0.00	0.00	0.00	9.21	10.30	0.00	9.21	9.76	17.48	6.50	0.00	3.79	8.67	0.00	9.76	2.17	1.63
T	5.84	0.00	3.19	0.00	0.00	13.41	0.00	5.84	0.00	0.00	0.00	7.44	24.70	0.00	21.38	0.00	18.19	0.00	0.00	0.00
N	0.00	9.72	20.37	21.12	0.00	28.60	6.73	0.00	0.00	0.00	0.00	0.00	0.00	0.00	7.48	0.00	0.00	0.00	5.98	0.00
Q	27.08	21.45	12.87	0.00	13.94	0.00	0.00	0.00	0.00	0.00	0.00	11.80	0.00	0.00	0.00	12.87	0.00	0.00	0.00	0.00
C	22.97	0.00	0.00	0.00	0.00	22.97	0.00	0.00	0.00	0.00	8.11	0.00	0.00	0.00	0.00	0.00	0.00	9.46	25.68	10.81
G	28.64	0.00	0.00	11.28	11.28	20.91	0.00	0.00	0.00	6.08	0.00	0.00	10.14	8.62	0.00	0.00	0.00	0.00	0.00	3.04
P	8.86	4.77	0.00	0.00	0.00	24.02	8.86	0.00	5.45	0.00	0.00	0.00	9.54	0.00	0.00	38.50	0.00	0.00	0.00	0.00
A	0.00	0.00	0.00	6.05	2.79	8.84	29.65	0.00	0.00	0.00	11.28	11.74	0.00	29.65	0.00	0.00	0.00	0.00	0.00	0.00
V	0.00	0.00	0.00	2.08	4.58	0.00	0.00	0.00	0.00	0.00	6.67	0.00	22.71	0.00	21.04	17.71	21.88	3.33	0.00	0.00
I	1.80	0.00	1.20	0.00	0.00	6.02	26.17	4.81	0.00	0.00	0.00	0.00	0.00	28.57	0.00	10.83	15.19	5.41	0.00	0.00
L	8.29	3.63	0.00	0.00	0.00	7.77	0.00	0.00	7.25	0.00	0.00	28.76	0.00	13.08	5.18	0.00	6.22	17.75	0.00	2.07
M	8.86	0.00	4.43	0.00	0.00	0.00	22.16	0.00	0.00	0.00	0.00	0.00	0.00	21.05	31.30	12.19	0.00	0.00	0.00	0.00
F	0.00	0.00	0.00	0.00	0.00	20.35	0.00	0.00	0.00	10.08	0.00	0.00	0.00	6.98	7.75	49.42	0.00	0.00	5.43	0.00
Y	0.00	35.31	0.00	5.65	0.00	10.17	0.00	7.91	0.00	33.05	0.00	0.00	0.00	0.00	0.00	0.00	0.00	7.91	0.00	0.00
W	42.86	0.00	0.00	0.00	0.00	7.14	0.00	0.00	0.00	25.00	10.71	0.00	0.00	0.00	0.00	14.29	0.00	0.00	0.00	0.00

Supplementary Table 7. Number of samples annotated to protein domain in MdrDB (Counts > 100).

Protein Domain	Counts
Protein kinase domain	39693
DNA-binding domain	18643
Retroviral matrix protein	4785
Bromodomain	4736
Sema domain	2779
Phosphatidylinositol 3-/4-kinase, catalytic domain	2272
Phosphatidylinositol 3-kinase, C2 domain	2242
Nuclear hormone receptor, ligand-binding domain	2211
Phosphatidylinositol 3-kinase Ras-binding domain	2140
Phosphoinositide 3-kinase, accessory domain	2047
Nicotinate phosphoribosyltransferase	1207
DNA topoisomerase I, DNA binding, eukaryotic-type	841
Kinesin motor domain	579
Receptor L-domain	413
Histone deacetylase domain	422
Dihydrofolate reductase domain	370
Thymidylate synthase/dCMP hydroxymethylase domain	347
FAT domain	222
POLO box domain	216
Lon protease, N-terminal domain	213
Peptidase M24	199
PI3Kdelta, catalytic domain	182
Frizzled/Smoothened, transmembrane domain	173
Prolyl 4-hydroxylase, alpha subunit	161
Growth factor receptor domain 4	127
Phosphatidylinositol 3-kinase, adaptor-binding domain	123
THIF-type NAD/FAD binding fold	106

Supplementary Table 8. Number of samples annotated to pharmacological mechanism categories in MdrDB.

Pharmacological Mechanism	Counts
Kinase Inhibitor	12077
HIV-1 Reverse Transcriptase Inhibitor	4483
Estrogen Agonist/Antagonist	1076
Topoisomerase Inhibitor	306
Folate Analog Metabolic Inhibitor	299
Dihydrofolate Reductase Inhibitor Antimalarial	276
Protease Inhibitor	260
Hedgehog Pathway Inhibitor	134
BCL-2 Inhibitor	126
Histone Deacetylase Inhibitor	101
Poly(ADP-Ribose) Polymerase Inhibitor	93
Neuraminidase Inhibitor	17
Dihydrofolate Reductase Inhibitor Antibacterial	10
Macrolide Antimicrobial	7
l-Thyroxine	3
Azole Antifungal	2
Nitrogen Binding Agent	2
Cholinergic Receptor Agonist	1
Retinoid	1
Cholinesterase Inhibitor	1

Supplementary Table 9. Overview of training dataset represented in three scenarios.

Scenario	Dataset	No. of samples	No. of resistant	No. of susceptible	No. of protein family
Scenario1	Platinum (no tyrosine kinase)	484	120	364	63
Scenario2	Platinum	649	154	495	77
Scenario3	MdrDB Coreset (single substitution)	3553	587	2996	147

Supplementary Note1: Model performance evaluation

In this section, we provide a comprehensive evaluation of 10 common machine learning models in several scenarios, and provide baseline prediction results on the MdrDB database. The corresponding code is available at https://github.com/tencent-quantum-lab/MdrDB.

The following are the details of the four main experimental scenarios:

- ***Scenario 1:* Evaluate the prediction performance of the machine learning methods on the MdrDB_CoreSet, with samples corresponding to single substitutions.**
  - ***Scenario 1.1:*** Randomly split the samples. Approximately 80% of the data is used as training samples, and the remaining 20% as test samples.
  - ***Scenario 1.2:*** 5-fold cross-validation. All data is randomly split into 5 folds, and then the machine learning method is trained on the 4 folds, while one fold is left to test the model. This process is repeated 5 times to obtain predictions for all data.
  - ***Scenario 1.3:*** Group 5-fold cross-validation (Uniprot ID). Samples are grouped according to Uniprot ID, and all data is randomly divided into 5 folds, where the same group does not appear in two different folds. The machine learning method is trained on the k - 1 folds, while one-fold is left to test the method. This process is repeated 5 times to obtain predictions for all data.
  - ***Scenario 1.4:*** 5-fold nested cross-validation (protein sequence). Protein sequences are obtained according to Uniprot ID and encoded by one-hot encoding. Then, the protein sequences were divided into 5 groups by k-nearest neighbors (KNN) clustering. We then use 5-fold nested cross-validation. At each iteration, the machine learning method is trained on the 4 folds with 4 groups, while one fold is left to test the method. This process is repeated 5 times to obtain predictions for all data.
  - ***Scenario 1.5:*** Group 5-fold cross-validation (drug name). Samples are grouped according to drug name, and all data is randomly divided into 5 folds, where the same group does not appear in two different folds. The machine learning method is trained on the 4 folds, while one-fold is left to test the method. This process is repeated 5 times to obtain predictions for all data.
  - ***Scenario 1.6:*** 5-fold nested cross-validation (SMILES). SMILES strings are first converted into molecular fingerprints, which are binary vectors that encode the presence or absence of certain substructures in the molecule. Pairwise Tanimoto similarity between fingerprints is then calculated as a

measure of similarity between molecules. Finally, the KNN clustering algorithm is used to group similar molecules into 5 clusters based on their pairwise similarities. We then use 5-fold nested cross-validation. At each iteration, the machine learning method is trained on the 4 folds with 4 groups, while one fold is left to test the method. This process is repeated 5 times to obtain predictions for all data.

- ***Scenario 1.7:*** 25-fold nested cross-validation (amino acid type). Amino acid type changes (from wild type to mutation) are extracted from the mutation information of the dataset. For instance, the mutation information of one data is "A256R", in which amino acid A belongs to the Hydrophobic group and amino acid R belongs to the Positve group. Then, the amino acid type of this data is "Hydrophobic_Positve". In the manuscript, the 20 amino acids are divided into five groups (i.e., positive, negative, polar, special cases, and hydrophobic). Thus, there are a total of 5*5=25 groups of amino acid changes before and after mutation. Grouping samples according to the amino acid type changes. The machine learning method is trained on the 24 folds, while one fold is left to test the method. This process is repeated 25 times to obtain predictions for all data.
- ***Scenario 1.8:*** 237-fold nested cross-validation (amino acid). Amino acid changes (from wild type to mutation) are extracted from the mutation information of the dataset. For instance, the mutation information of one data is "A256R", and the amino acid change of this data is "A_R". Grouping samples according to the amino acid changes (237 groups). The machine learning method is trained on the 236 folds, while one fold is left to test the method. This process is repeated 237 times to obtain predictions for all data.

- ***Scenario 2:*** **Evaluate the prediction performance of the machine learning methods on the MdrDB_CoreSet, with samples corresponding to multiple substitutions.**
  - ***Scenario 2.1:*** Randomly split the samples. Approximately 80% of the data is used as training samples, with the remaining 20% as test samples.
  - ***Scenario 2.2:*** 5-fold cross-validation. All data is randomly split into 5 folds, and then the machine learning method is trained on the 4 folds, while one fold is left to test the model. This process is repeated 5 times to obtain predictions for all data.

- ***Scenario 3:*** **Evaluate the prediction performance of the machine learning methods on the MdrDB_CoreSet. The models are trained on the single**

substitution samples and tested on the multiple substitutions/(deletion, indel, insertion, complex) mutation samples.

- ***Scenario 3.1:*** Train on single substitution, and test on multiple substitutions.
- **Scenario 3.2:** Train on single substitution, and test on (deletion, indel, insertion, complex) mutations.

- ***Scenario 4:*** **Evaluate the prediction performance of the machine learning methods on the MdrDB_CoreSet. The models are trained on the single substitution samples, fine-tuned on 80% of the data from multiple substitutions/deletion mutations, and tested on the remaining 20% of the multiple substitutions/deletion mutations data.**
  - ***Scenario 4.1:*** Train on single substitution; fine-tune, and test on multiple substitutions.
  - ***Scenario 4.2:*** Train on single substitutions; fine-tune and test on (deletion, indel, insertion, complex) mutations.

Baselines: As mentioned in the manuscript, four families of methods were used to evaluate the drug resistance prediction performance. The first family consist of tree-based methods, including decision tree (DecisionTree), random forest (RandomForest), and extremely randomized regression trees (ExtraTrees). The second family comprise linear-based methods: support vector regression (SVR), elastic net linear regression (Elastic Net), and lasso regression (Lasso). The third family of baselines is ensemble-based methods including bagging regressor (Bagging), AdaBoost, and gradient boosting (GradienBoost). The fourth family is neural network-based methods such as multi-layer perceptron (MLP).

Evaluation metrics: Root Mean Square Error (RMSE), Pearson correlation coefficient (Pears), and the area under the precision-recall curve (AUPRC) were used to evaluate model performance. Consistent with the previous work, resistant mutations are defined as the affinity changes for mutants by least 10-fold, i.e., $\Delta\Delta G_{exp} > 1.36$ kcal mol^{-1}.

The following are the specific experimental results:

Scenario 1.1: Randomly split the samples (single substitution).

In scenario 1.1, we report the RMSE, Pearson, and AUPRC averaged over 5 repetitions for each machine learning method in Supplementary Table 10. Supplementary Figure 10 shows the scatter plots of the experimental versus calculated ΔΔG values in one experiment. It can be observed that ExtraTrees outperforms other machine learning

methods on MdrDB_CoreSet (single substitution). Furthermore, most of the tree-based and ensemble-based methods obtain better prediction performance than linear-based and neural network-based methods in this scenario.

Supplementary Table 10. Test prediction performance on MdrDB_CoreSet (single substitution). Mean performance (±std) over 5 repetitions are reported. The best is highlighted in bold.

Methods	RMSE	Pearson	AUPRC
**MLP**	2.622 ± 1.241	0.048 ± 0.049	0.224 ± 0.027
**DecisionTree**	1.595 ± 0.101	0.314 ± 0.081	0.312 ± 0.02
**AdaBoost**	1.516 ± 0.097	0.107 ± 0.07	0.248 ± 0.026
**Lasso**	1.343 ± 0.125	0.108 ± 0.041	0.237 ± 0.018
**ElasticNet**	1.341 ± 0.127	0.126 ± 0.044	0.244 ± 0.022
**SVR**	1.335 ± 0.122	0.152 ± 0.031	0.261 ± 0.016
**GradientBoost**	1.194 ± 0.09	0.473 ± 0.046	0.386 ± 0.033
**Bagging**	1.166 ± 0.116	0.518 ± 0.065	0.44 ± 0.05
**RandomForest**	1.159 ± 0.116	0.528 ± 0.055	0.438 ± 0.051
**ExtraTrees**	**1.121 ± 0.135**	**0.56 ± 0.068**	**0.483 ± 0.039**

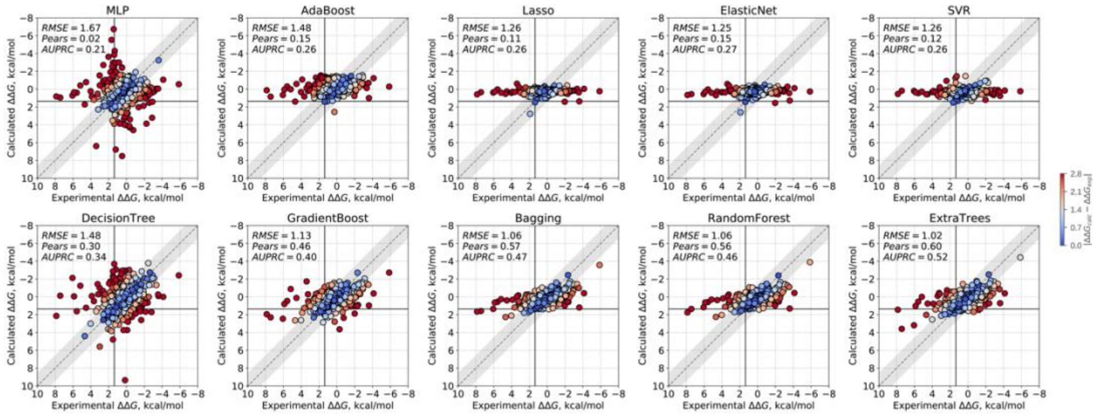

Supplementary Figure 10. Scatter plots of the experimental versus calculated ΔΔG values in Scenario 1.1. Each ΔΔG estimate is color-coded according to its absolute error w.r.t. the experimental ΔΔG value; at 300 K, a 1.4 kcal mol^{-1} error corresponds

to a 10-fold error in the Kd change and 2.8 kcal mol^{-1} error corresponds to a 100-fold error in the Kd change.

Scenario 1.2: 5-fold cross-validation (single substitution).

Supplementary Table 11 shows the mean and standard deviation for performance on MdrDB_CoreSet (single substitution) under 5-fold cross-validation, and Supplementary Figure 11 plots the scatter plots of the experimental versus calculated ΔΔG values in Scenario 1.2. It can be observed that ExtraTrees outperforms other machine learning methods on MdrDB_CoreSet (single substitution). Furthermore, consistent with Scenario 1.1, most of the tree-based and ensemble-based methods obtain better prediction performance than linear-based and neural network-based methods in this scenario.

Supplementary Table 11. Prediction performance obtained with 5-fold cross-validation on MdrDB_CoreSet (single substitution). Mean and standard deviation are reported. The best is highlighted in **bold**.

Methods	RMSE	Pearson	AUPRC
**MLP**	1.951 ± 0.528	0.03 ± 0.034	0.221 ± 0.036
**DecisionTree**	1.608 ± 0.078	0.352 ± 0.101	0.316 ± 0.012
**AdaBoost**	1.501 ± 0.141	0.172 ± 0.102	0.234 ± 0.014
**Lasso**	1.322 ± 0.137	0.114 ± 0.043	0.226 ± 0.026
**ElasticNet**	1.319 ± 0.139	0.132 ± 0.049	0.231 ± 0.022
**SVR**	1.302 ± 0.14	0.203 ± 0.038	0.27 ± 0.029
**GradientBoost**	1.186 ± 0.133	0.48 ± 0.044	0.407 ± 0.036
**Bagging**	1.147 ± 0.131	0.523 ± 0.057	0.467 ± 0.046
**RandomForest**	1.146 ± 0.133	0.523 ± 0.052	0.463 ± 0.046
**ExtraTrees**	**1.132 ± 0.146**	**0.533 ± 0.064**	**0.487 ± 0.038**

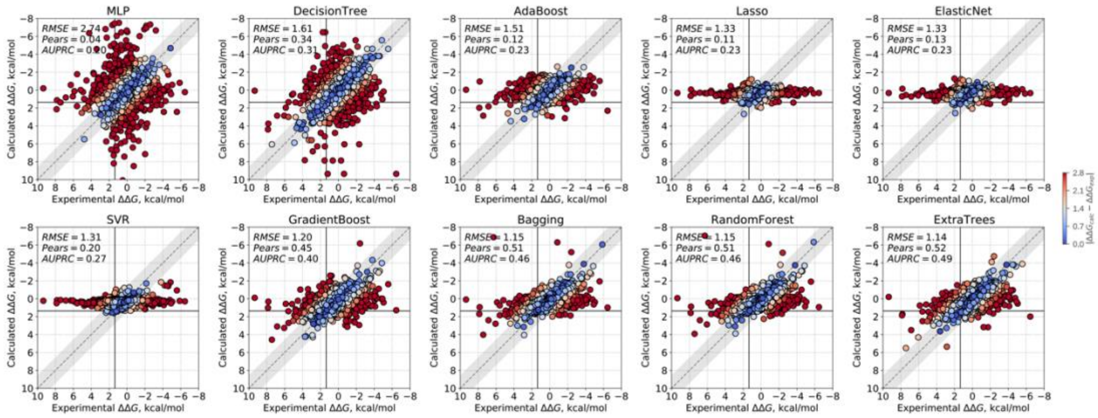

Supplementary Figure 11. Scatter plots of the experimental versus calculated ΔΔG values in Scenario 1.2. Each ΔΔG estimate is color-coded according to its absolute error w.r.t. the experimental ΔΔG value; at 300 K, the 1.4 kcal mol^{-1} error corresponds to a 10-fold error in the Kd change and 2.8 kcal mol^{-1} error corresponds to a 100-fold error in the Kd change.

Scenario 1.3: Group 5-fold cross-validation (Uniprot ID).

In Supplementary Table 12, we present the mean and standard deviation for performance on MdrDB_CoreSet (single substitution) using group 5-fold cross-validation based on Uniprot ID, and Supplementary Figure 12 displays the scatter plots of the experimental versus calculated ΔΔG values. We can clearly see that the machine learning methods perform relatively well in terms of RMSE, but their performance in terms of correlation and classification ability is significantly worse than that in Scenario 1.2. For example, SVR demonstrates a weak correlation (Pearson=0.062 ± 0.04) and poor classification performance (AUPRC=0.218 ± 0.038). A possible explanation for this outcome is that, in this scenario, the same protein group does not appear in two different folds, and the performance of machine learning methods may be degraded when they are trained on unseen protein groups (i.e., on unrelated data).

Supplementary Table 12. Prediction performance obtained with group 5-fold cross-validation based on Uniprot ID on MdrDB_CoreSet (single substitution). Mean and standard deviation are reported. The best is highlighted in **bold**.

Methods	RMSE	Pearson	AUPRC
**MLP**	3.495 ± 3.392	0.001 ± 0.092	0.201 ± 0.037
**DecisionTree**	2.081 ± 0.258	-0.009 ± 0.058	0.17 ± 0.037
**AdaBoost**	1.532 ± 0.164	0.028 ± 0.098	0.205 ± 0.04

**GradientBoost**	1.433 ± 0.21	0.036 ± 0.066	0.216 ± 0.039
**RandomForest**	1.389 ± 0.229	-0.008 ± 0.055	0.209 ± 0.021
**Bagging**	1.386 ± 0.227	-0.001 ± 0.068	0.218 ± 0.022
**ExtraTrees**	1.359 ± 0.234	0.051 ± 0.063	0.222 ± 0.025
**ElasticNet**	1.356 ± 0.243	0.008 ± 0.087	0.194 ± 0.033
**Lasso**	1.35 ± 0.246	-0.003 ± 0.067	0.19 ± 0.036
**SVR**	**1.327 ± 0.243**	**0.062 ± 0.04**	**0.218 ± 0.038**

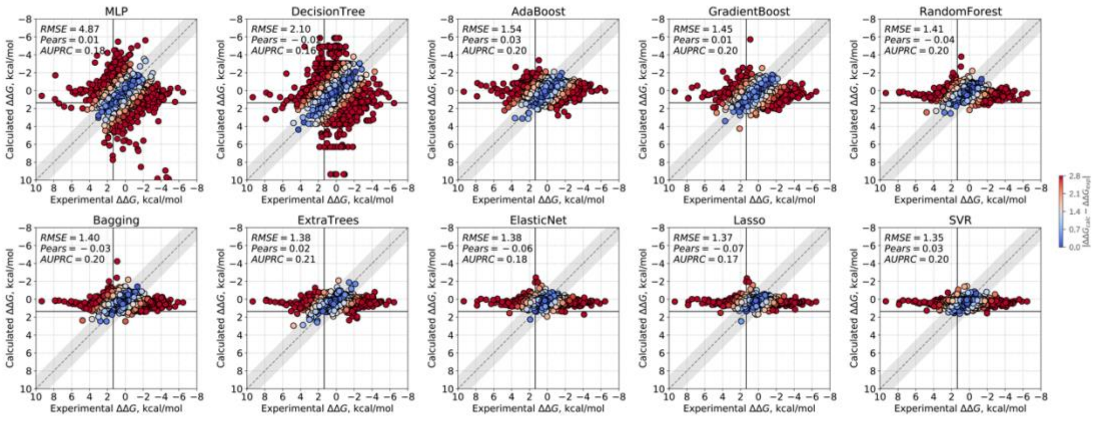

Supplementary Figure 12. Scatter plots of the experimental versus calculated ΔΔG values in Scenario 1.3. Each ΔΔG estimate is color-coded according to its absolute error w.r.t. the experimental ΔΔG value; at 300 K, the 1.4 kcal mol^{-1} error corresponds to a 10-fold error in the Kd change and 2.8 kcal mol^{-1} error corresponds to a 100-fold error in the Kd change.

Scenario 1.4: 5-fold nested cross-validation (protein sequence).

To further evaluate the ability of machine learning to predict drug resistance more broadly across protein classes, we calculated and clustered the similarity of protein sequences in MdrDB_CoreSet, divided them into five groups, and performed 5-fold nested cross-validation. Supplementary Table 13 shows the prediction performance of the machine learning methods, and Supplementary Figure 13 displays the scatter plots of the experimental versus calculated ΔΔG values. Although SVR achieves the best result among all competing methods, it has relatively poor RMSE (1.408 ± 0.291), weak correlation (Pearson=0.072 ± 0.034), and poor classification performance (AUPRC=0.24 ± 0.05) in this scenario. These results indicate that the generalization

performance of the current machine learning methods is poor when predicting drug-resistant mutations in protein families not seen in the training set.

Supplementary Table 13. Prediction performance obtained with 5-fold nested cross-validation (samples are clustered according to the similarity of amino acid sequences of proteins and are divided into 5 groups). Mean and standard deviation are reported. The best is highlighted in **bold**.

Methods	RMSE	Pearson	AUPRC
**MLP**	2.948 ± 0.813	-0.016 ± 0.036	0.202 ± 0.067
**DecisionTree**	1.998 ± 0.26	-0.031 ± 0.085	0.188 ± 0.053
**AdaBoost**	1.601 ± 0.27	0.072 ± 0.068	0.227 ± 0.075
**GradientBoost**	1.523 ± 0.3	-0.013 ± 0.075	0.198 ± 0.07
**ElasticNet**	1.453 ± 0.278	-0.075 ± 0.098	0.196 ± 0.077
**Lasso**	1.44 ± 0.28	-0.052 ± 0.097	0.195 ± 0.078
**ExtraTrees**	1.437 ± 0.283	0.003 ± 0.063	0.199 ± 0.063
**Bagging**	1.437 ± 0.29	0.002 ± 0.106	0.211 ± 0.085
**RandomForest**	1.436 ± 0.288	0.002 ± 0.096	0.207 ± 0.086
**SVR**	**1.408 ± 0.291**	**0.072 ± 0.034**	**0.24 ± 0.05**

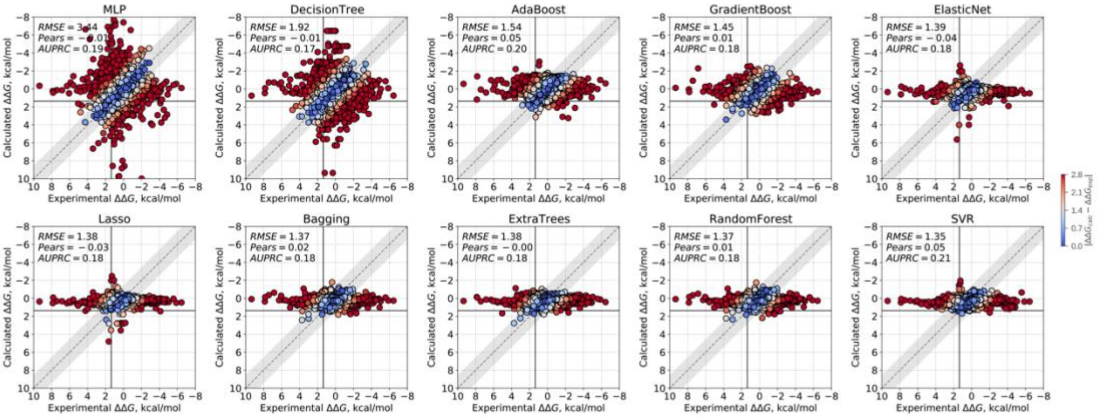

Supplementary Figure 13. Scatter plots of the experimental versus calculated ΔΔG values in Scenario 1.4. Each ΔΔG estimate is color-coded according to its absolute error w.r.t. the experimental ΔΔG value; at 300 K, the 1.4 kcal mol^{-1} error corresponds

to a 10-fold error in the Kd change and 2.8 kcal mol^{-1} error corresponds to a 100-fold error in the Kd change.

Scenario 1.5: Group 5-fold cross-validation (drug name).

To explore the ability of machine learning methods to predict drug resistance from a ligand perspective, we conduct group 5-fold cross-validation based on drug name on MdrDB_CoreSet. As shown in Supplementary Table 14, ExtraTrees obtains the best prediction performance, however, it has poor performance in terms of correlation (Pearson=0.161 ± 0.075) and classification ability (AUPRC=0.3 ± 0.06). In this scenario, the same ligand group does not appear in two different folds, which may be the reason why the prediction ability of the machine learning methods degrades when they are trained on unseen ligand groups.

Supplementary Table 14. Prediction performance obtained with group 5-fold cross-validation based on drug name on MdrDB_CoreSet (single substitution). Mean and standard deviation are reported. The best is highlighted in bold.

Methods	RMSE	Pearson	AUPRC
**MLP**	4.684 ± 4.267	0.036 ± 0.093	0.232 ± 0.052
**DecisionTree**	1.859 ± 0.207	0.039 ± 0.056	0.188 ± 0.052
**AdaBoost**	1.562 ± 0.145	0.024 ± 0.044	0.213 ± 0.045
**GradientBoost**	1.419 ± 0.213	0.075 ± 0.04	0.235 ± 0.035
**ElasticNet**	1.358 ± 0.214	-0.018 ± 0.02	0.188 ± 0.035
**Lasso**	1.351 ± 0.213	-0.022 ± 0.021	0.188 ± 0.034
**Bagging**	1.334 ± 0.212	0.115 ± 0.048	0.274 ± 0.052
**SVR**	1.331 ± 0.215	0.076 ± 0.039	0.217 ± 0.048
**RandomForest**	1.328 ± 0.21	0.125 ± 0.045	0.278 ± 0.057
**ExtraTrees**	**1.327 ± 0.205**	**0.161 ± 0.075**	**0.3 ± 0.06**

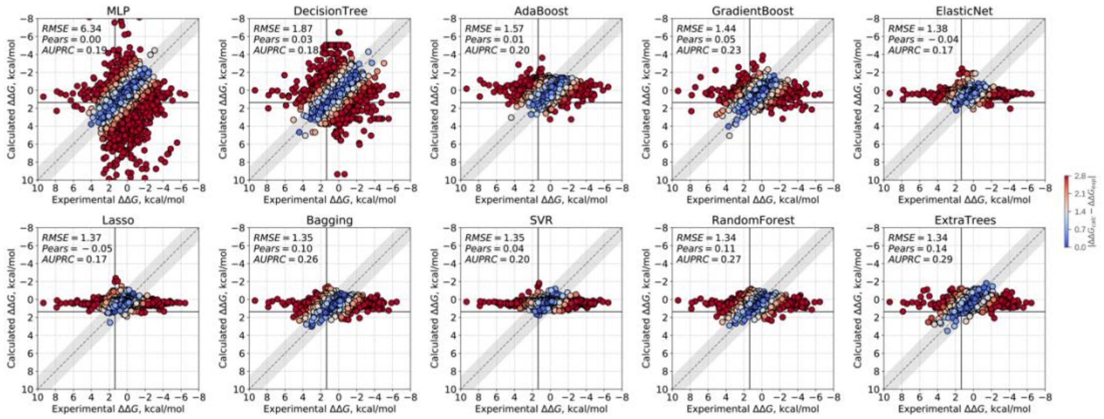

Supplementary Figure 14. Scatter plots of the experimental versus calculated ΔΔG values in Scenario 1.5. Each ΔΔG estimate is color-coded according to its absolute error w.r.t. the experimental ΔΔG value; at 300 K, the 1.4 kcal mol^{-1} error corresponds to a 10-fold error in the Kd change and 2.8 kcal mol^{-1} error corresponds to a 100-fold error in the Kd change.

Scenario 1.6: 5-fold nested cross-validation (SMILES).

To further explore the prediction ability of the machine learning methods to predict drug resistance more broadly across ligands, we calculated and clustered the similarity of SMILES of ligand in MdrDB_CoreSet, divided them into five groups, and performed 5-fold nested cross-validation. Supplementary Table 15 shows the prediction performance of the machine learning methods, and Supplementary Figure 15 displays the scatter plots of the experimental versus calculated ΔΔG values. Although RandomForest obtains the optimal results in terms of RMSE (1.181 ± 0.244), it has weak correlation (Pearson=0.016 ± 0.056), and poor classification performance (AUPRC=0.191 ± 0.133). This result indicates that the generalization performance of current machine learning methods has poor performance when predicting drug-resistant mutations in ligand classes not seen in the training set.

Supplementary Table 15. Prediction performance obtained with 5-fold nested cross-validation (the samples are clustered according to the similarity of SMILES of drug and are divided into 5 groups). Mean and standard deviation are reported. The best is highlighted in **bold**.

Methods	RMSE	Pearson	AUPRC
**MLP**	2.615 ± 1.852	0.039 ± 0.044	0.183 ± 0.089
**DecisionTree**	1.703 ± 0.203	0.022 ± 0.031	0.234 ± 0.089
**AdaBoost**	1.395 ± 0.252	-0.07 ± 0.043	0.194 ± 0.13

**GradientBoost**	1.233 ± 0.278	0.07 ± 0.109	0.191 ± 0.123
**Bagging**	1.192 ± 0.23	0.018 ± 0.054	0.186 ± 0.123
**ElasticNet**	1.19 ± 0.254	-0.019 ± 0.046	0.173 ± 0.099
**Lasso**	1.189 ± 0.249	-0.034 ± 0.039	0.177 ± 0.097
**ExtraTrees**	1.186 ± 0.244	0.042 ± 0.121	0.216 ± 0.151
**SVR**	1.184 ± 0.232	0.047 ± 0.058	0.181 ± 0.123
**RandomForest**	**1.181 ± 0.244**	**0.016 ± 0.056**	**0.191 ± 0.133**

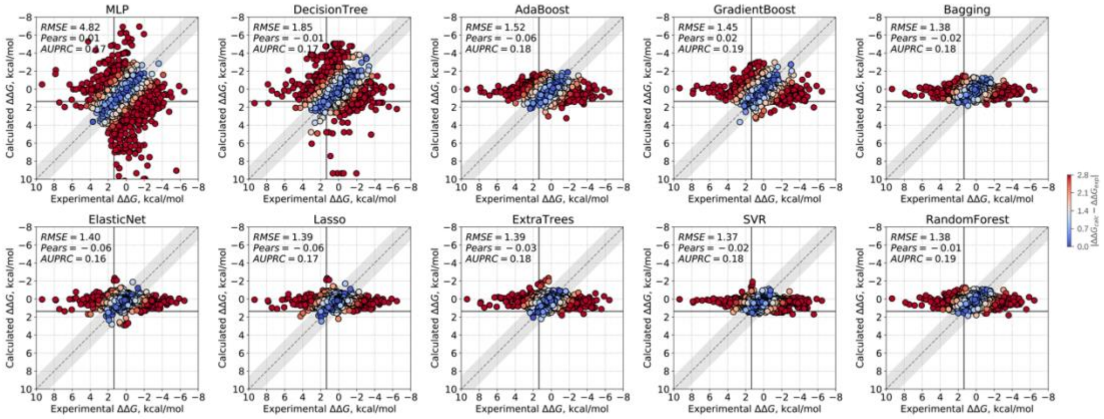

Supplementary Figure 15. Scatter plots of the experimental versus calculated ΔΔG values in Scenario 1.6. Each ΔΔG estimate is color-coded according to its absolute error w.r.t. the experimental ΔΔG value; at 300 K, the 1.4 kcal mol^{-1} error corresponds to a 10-fold error in the Kd change and 2.8 kcal mol^{-1} error corresponds to a 100-fold error in the Kd change.

Scenario 1.7: 25-fold nested cross-validation (amino acid type).

To evaluate the capability of machine learning in predicting drug resistance across mutation aspects, we performed 25-fold cross-validation based on the type of amino acid change from wild type to mutation. Supplementary Table 16 shows the prediction performance of the machine learning methods, and Supplementary Figure 16 displays the scatter plots of the experimental versus calculated ΔΔG values in this scenario. ExtraTrees, RandomForest, and Bagging achieve similar prediction results, with relatively good RMSE and correlation, but poor classification performance.

Supplementary Table 16. Prediction performance was obtained with 25-fold nested cross-validation according to the amino acid change type from wild type to mutation. Mean and standard deviation are reported. The best is highlighted in **bold**.

Methods	RMSE	Pearson	AUPRC
**MLP**	1.823 ± 0.599	0.067 ± 0.164	0.23 ± 0.1
**DecisionTree**	1.701 ± 0.497	0.263 ± 0.154	0.293 ± 0.109
**AdaBoost**	1.615 ± 0.454	0.081 ± 0.195	0.241 ± 0.1
**Lasso**	1.364 ± 0.48	0.074 ± 0.169	0.228 ± 0.113
**ElasticNet**	1.359 ± 0.482	0.113 ± 0.146	0.238 ± 0.119
**SVR**	1.344 ± 0.474	0.178 ± 0.13	0.261 ± 0.126
**GradientBoost**	1.204 ± 0.392	0.482 ± 0.168	0.392 ± 0.128
**Bagging**	1.157 ± 0.395	0.541 ± 0.164	0.473 ± 0.17
**RandomForest**	1.154 ± 0.396	**0.544 ± 0.167**	0.459 ± 0.165
**ExtraTrees**	**1.151 ± 0.413**	0.542 ± 0.154	**0.475 ± 0.169**

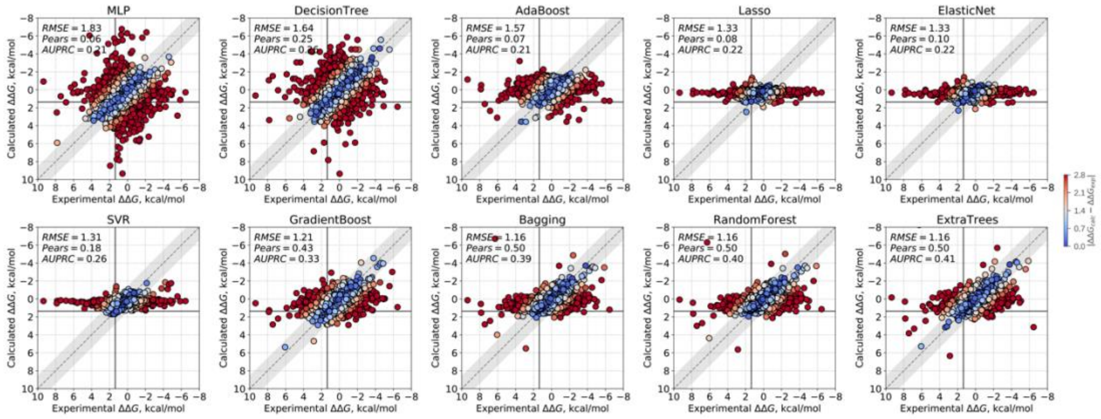

Supplementary Figure 16. Scatter plots of the experimental versus calculated ΔΔG values in Scenario 1.7. Each ΔΔG estimate is color-coded according to its absolute error w.r.t. the experimental ΔΔG value; at 300 K, the 1.4 kcal mol^{-1} error corresponds to a 10-fold error in the Kd change and 2.8 kcal mol^{-1} error corresponds to a 100-fold error in the Kd change.

Scenario 1.8: 237-fold nested cross-validation (amino acid).

We conducted 237-fold nested cross-validation based on the amino acid change from wild type to mutation. As shown in Supplementary Table 17, ExtraTrees achieves the best results compared with other competing methods. It performs relatively well in RMSE (1.142 ± 0.053) and correlation (Pearson = 0.518 ± 0.061), but is poor in classification ability (AUPRC = 0.449 ± 0.071), which is similar to the results in Scenario 1.7. Supplementary Figure 17 displays the scatter plots of the experimental versus calculated ΔΔG values in this scenario.

Supplementary Table 17. Prediction performance obtained with 237-fold nested cross-validation according to the amino acid change from wild type to mutation. Mean and standard deviation are reported. The best is highlighted in **bold**.

Methods	RMSE	Pearson	AUPRC
**MLP**	6.535 ± 6.663	0.066 ± 0.038	0.222 ± 0.019
**DecisionTree**	1.603 ± 0.04	0.261 ± 0.112	0.271 ± 0.058
**AdaBoost**	1.543 ± 0.092	0.141 ± 0.106	0.235 ± 0.043
**Lasso**	1.325 ± 0.109	0.11 ± 0.016	0.235 ± 0.023
**ElasticNet**	1.323 ± 0.108	0.128 ± 0.024	0.243 ± 0.025
**SVR**	1.305 ± 0.108	0.199 ± 0.032	0.267 ± 0.032
**GradientBoost**	1.187 ± 0.057	0.461 ± 0.051	0.351 ± 0.067
**Bagging**	1.153 ± 0.065	0.509 ± 0.065	0.423 ± 0.07
**RandomForest**	1.15 ± 0.065	0.513 ± 0.063	0.424 ± 0.073
**ExtraTrees**	**1.142 ± 0.053**	**0.518 ± 0.061**	**0.449 ± 0.071**

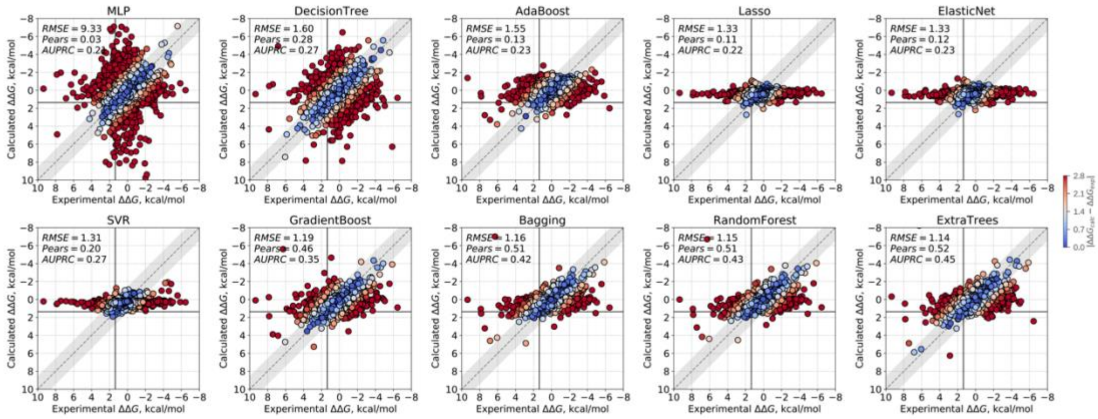

Supplementary Figure 17. Scatter plots of the experimental versus calculated ΔΔG values in Scenario 1.8. Each ΔΔG estimate is color-coded according to its absolute error w.r.t. the experimental ΔΔG value; at 300 K, the 1.4 kcal mol^{-1} error corresponds to a 10-fold error in the Kd change and 2.8 kcal mol^{-1} error corresponds to a 100-fold error in the Kd change.

Scenario 2.1: Randomly split the samples (multiple substitutions).

In scenario 2.1, we report the RMSE, Pearson, and AUPRC averaged over 5 repetitions for each machine learning method in Supplementary Table 18. Supplementary Figure 18 shows the scatter plots of the experimental versus calculated ΔΔG values in one experiment. It can be observed that ExtraTrees outperforms other machine learning methods on MdrDB_CoreSet (multiple substitutions). Furthermore, most of the tree-based and ensemble-based methods obtain better prediction performance than linear-based and neural network-based methods in this scenario.

Supplementary Table 18. Test prediction performance on MdrDB_CoreSet (multiple substitutions). Mean performance (±std) over 5 repetitions are reported. The best is highlighted in **bold**.

Methods	RMSE	Pearson	AUPRC
**MLP**	2.427 ± 0.384	0.121 ± 0.07	0.188 ± 0.053
**DecisionTree**	1.537 ± 0.231	0.319 ± 0.189	0.386 ± 0.215
**ElasticNet**	1.347 ± 0.093	0.205 ± 0.059	0.315 ± 0.066
**Lasso**	1.339 ± 0.092	0.183 ± 0.06	0.322 ± 0.074
**SVR**	1.298 ± 0.09	0.179 ± 0.089	0.359 ± 0.045

**AdaBoost**	1.206 ± 0.066	0.413 ± 0.046	0.514 ± 0.075
**GradientBoost**	1.162 ± 0.119	0.488 ± 0.075	0.516 ± 0.109
**Bagging**	1.136 ± 0.118	0.513 ± 0.083	0.593 ± 0.14
**RandomForest**	1.134 ± 0.111	0.513 ± 0.08	0.583 ± 0.15
**ExtraTrees**	**1.11 ± 0.129**	**0.547 ± 0.077**	**0.625 ± 0.151**

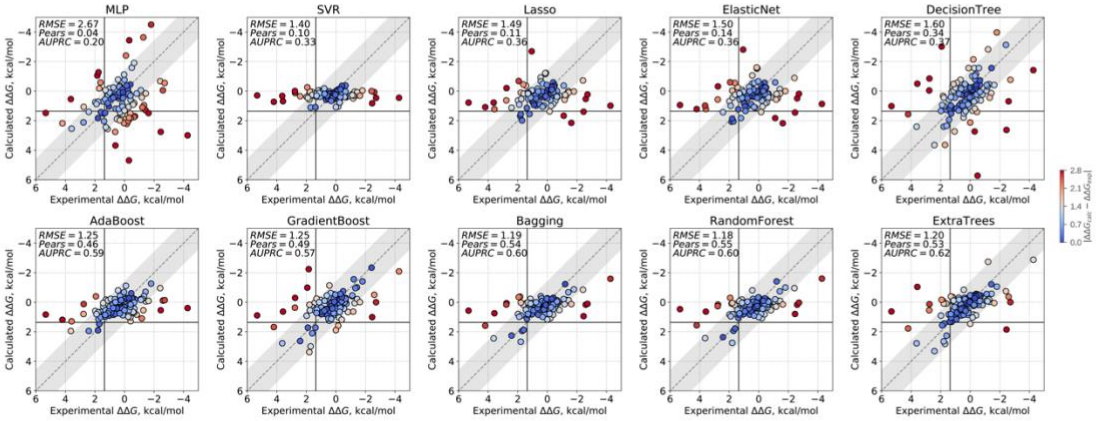

Supplementary Figure 18. Scatter plots of the experimental versus calculated ΔΔG values in Scenario 2.1. Each ΔΔG estimate is color-coded according to its absolute error w.r.t. the experimental ΔΔG value; at 300 K, the 1.4 kcal mol^{-1} error corresponds to a 10-fold error in the Kd change and 2.8 kcal mol^{-1} error corresponds to a 100-fold error in the Kd change.

Scenario 2.2: 5-fold cross-validation (multiple substitutions).

Supplementary Table 19 shows the mean and standard deviation for performance on MdrDB_CoreSet (multiple substitutions) under 5-fold cross-validation, and Supplementary Figure 19 plots the scatter plots of the experimental versus calculated ΔΔG values in this Scenario. ExtraTrees outperforms other machine learning methods on MdrDB_Coreset (multiple substitutions). Consistent with Scenario 2.1, the tree-based and ensemble-based methods obtain better prediction performance than linear-based and neural network-based methods in this scenario.

Supplementary Table 19. Prediction performance obtained with 5-fold cross-validation on MdrDB_CoreSet (multiple substitutions). Mean and standard deviation are reported. The best is highlighted in **bold**.

Methods	RMSE	Pearson	AUPRC

**MLP**	5.37 ± 4.409	0.083 ± 0.05	0.211 ± 0.067
**DecisionTree**	1.606 ± 0.118	0.287 ± 0.09	0.306 ± 0.043
**ElasticNet**	1.324 ± 0.099	0.242 ± 0.07	0.311 ± 0.113
**Lasso**	1.316 ± 0.114	0.221 ± 0.078	0.297 ± 0.085
**SVR**	1.307 ± 0.105	0.155 ± 0.1	0.31 ± 0.057
**GradientBoost**	1.206 ± 0.134	0.443 ± 0.054	0.505 ± 0.081
**AdaBoost**	1.204 ± 0.123	0.428 ± 0.076	0.488 ± 0.078
**RandomForest**	1.144 ± 0.167	0.511 ± 0.095	0.544 ± 0.069
**Bagging**	1.137 ± 0.164	0.506 ± 0.097	0.541 ± 0.073
**ExtraTrees**	**1.108 ± 0.179**	**0.539 ± 0.101**	**0.547 ± 0.061**

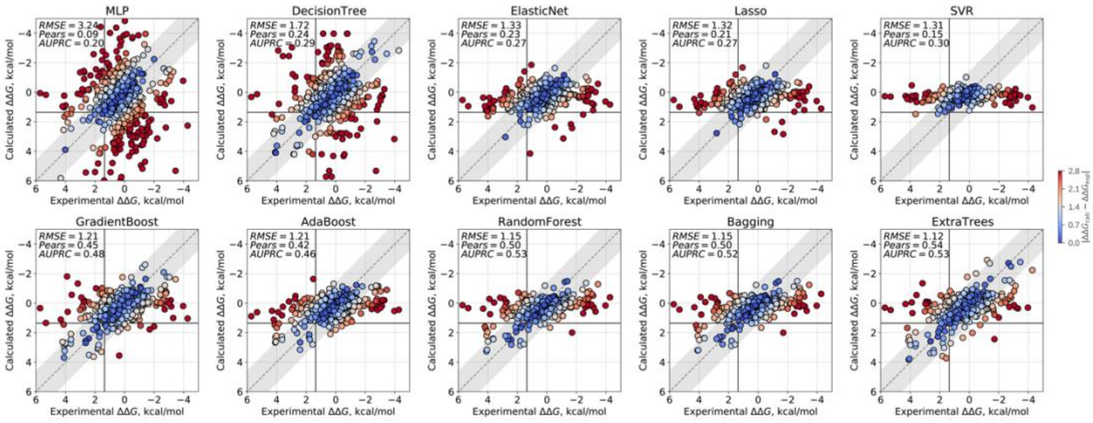

Supplementary Figure 19. Scatter plots of the experimental versus calculated ΔΔG values in Scenario 2.2. Each ΔΔG estimate is color-coded according to its absolute error w.r.t. the experimental ΔΔG value; at 300 K, the 1.4 kcal mol^{-1} error corresponds to a 10-fold error in the Kd change and 2.8 kcal mol^{-1} error corresponds to a 100-fold error in the Kd change.

Scenario 3.1: Training on the single substitution, and test on the multiple substitutions.

In Scenario 3.1, the machine learning methods are trained on MdrDB_CoreSet (single substitution), and then tested on the MdrDB_CoreSet (multiple substitutions). We aimed to assess whether the model can be extrapolated to multiple substitution mutation. Supplementary Table 20 shows the test prediction ability of the machine learning methods in this scenario, and the corresponding scatter plot of the experimental versus calculated ΔΔG values of multiple substitution samples is displayed in Supplementary Figure 20. ExtraTrees obtains the best performance in this scenario, although can only obtain a relatively weak correlation (Pearson=0.272) and poor classification ability (AUPRC=0.337). This indicates that the robustness of the current machine learning method in the task of predicting drug resistance from single substitution mutations extrapolate to multiple point mutations still needs to be improved.

Supplementary Table 20. Test prediction performance on MdrDB_CoreSet (multiple substitutions). The best is highlighted in **bold**.

Methods	RMSE	Pearson	AUPRC
**MLP**	7.119	-0.018	0.178
**DecisionTree**	2.517	0.041	0.17
**GradientBoost**	1.591	0.121	0.274
**RandomForest**	1.497	0.046	0.267
**Bagging**	1.487	0.05	0.257
**AdaBoost**	1.469	0.031	0.175
**SVR**	1.331	0.067	0.164
**ElasticNet**	1.327	0.132	0.18
**Lasso**	1.324	0.12	0.166
**ExtraTrees**	**1.292**	**0.272**	**0.337**

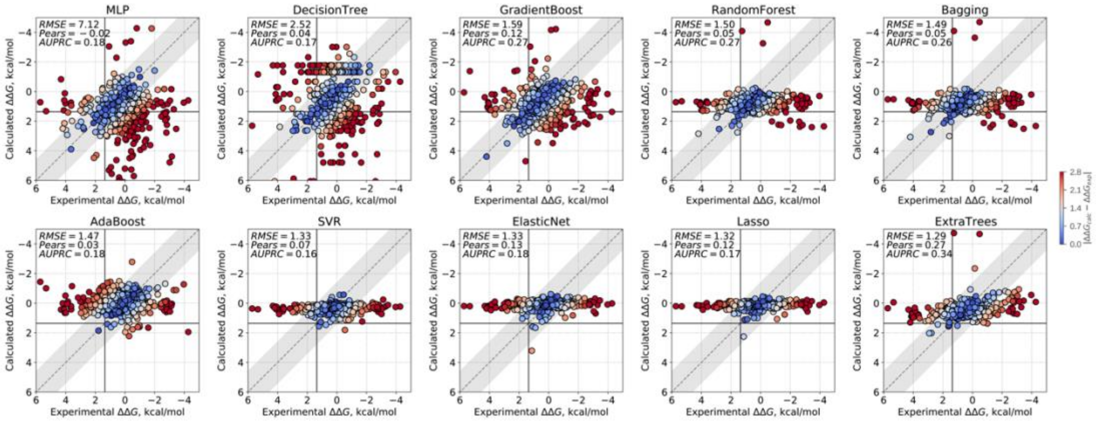

Supplementary Figure 20. Scatter plots of the experimental versus calculated ΔΔG values in Scenario 3.1. Each ΔΔG estimate is color-coded according to its absolute error w.r.t. the experimental ΔΔG value; at 300 K, the 1.4 kcal mol^{-1} error corresponds to a 10-fold error in the Kd change and 2.8 kcal mol^{-1} error corresponds to a 100-fold error in the Kd change.

Scenario 3.2: Training on the single substitution, test on the (deletion+indel+insertion+complex) mutation.

In Scenario 3.2, the machine learning methods are trained on MdrDB_CoreSet (single substitution) and then tested on the MdrDB_CoreSet (complex mutations, i.e., deletion, indel, insertion, and complex). Since the number of complex mutation samples in MdrDB_CoreSet is small, accounting for only 1.3% of all samples, samples of various complex mutation types (deletion, insertion, etc.) are used as the test set in this scenario to evaluate the extrapolation ability of the model to the complex mutation. As shown in Supplementary Table 21, AdaBoost achieves the best prediction performance in this scenario, but it obtains a relatively poor prediction ability (RMSE=1.375) and weak correlation (Pearson=0.261). This indicates that current machine learning methods still have major challenges to overcome in the task of inferring from single substitution mutations to predicting drug resistance caused by complex mutations. Because the number of drug-resistant samples in complex mutations is small (sometimes the test set is all susceptible samples), the classification ability of the model is not considered here.

Supplementary Table 21. Test prediction performance on MdrDB_CoreSet (deletion+indel+insertion+complex). The best is highlighted in **bold**.

Methods	RMSE	Pearson
**MLP**	17.754	0.137
**DecisionTree**	2.8	-0.196
**Bagging**	1.994	-0.112
**RandomForest**	1.99	-0.086
**SVR**	1.811	-0.179
**GradientBoost**	1.654	0.095
**Lasso**	1.641	0.036
**ElasticNet**	1.62	0.039
**ExtraTrees**	1.609	0.136
**AdaBoost**	**1.375**	**0.261**

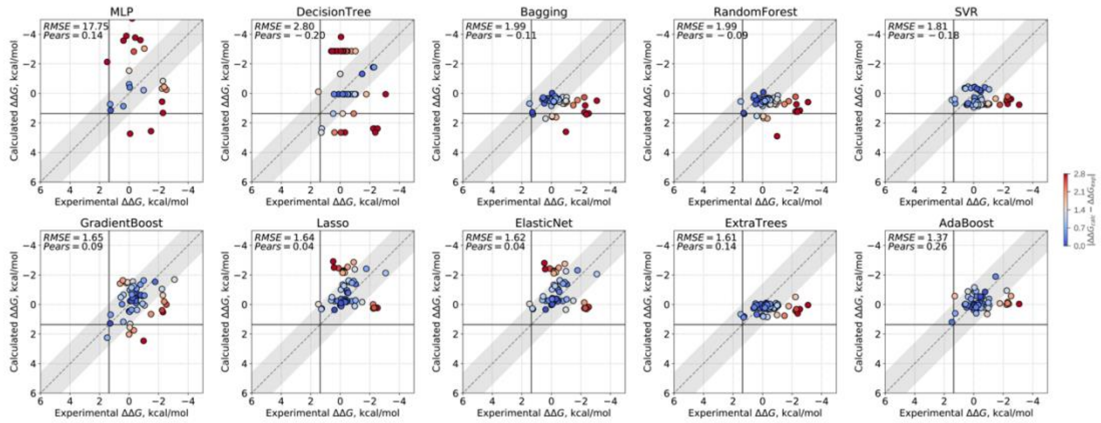

Supplementary Figure 21. Scatter plots of the experimental versus calculated ΔΔG values in Scenario 3.2. Each ΔΔG estimate is color-coded according to its absolute error w.r.t. the experimental ΔΔG value; at 300 K, the 1.4 kcal mol^{-1} error corresponds to a 10-fold error in the Kd change and 2.8 kcal mol^{-1} error corresponds to a 100-fold error in the Kd change.

Scenario 4.1: Training on the single substitution, fine-tuning on the multiple substitutions (fine-tune : test = 8 : 2).

To further explore the prediction ability of the current machine learning methods for drug-resistance prediction problems, we conduct experiments on MdrDB_CoreSet in a fine-tuned manner. Specifically, the machine learning methods are trained on the MdrDB_CoreSet (single substitution), then the model parameters are fine-tuned on approximately 80% of the samples in MdrDB_CoreSet (multiple substitutions), and then tested on the remaining multiple substitution samples. Supplementary Table 22 shows the average prediction performance of all the competing machine learning methods over 5 repetitions, and the scatter plots of the experimental versus calculated ΔΔG values in one experiment are displayed in Supplementary Figure 22. ExtraTrees achieves the best performance in this scenario. Compared with the results obtained in Scenario 2.1, pre-training the model on MdrDB_CoreSet (single replacement) helps to improve the resistance prediction ability of the model on multiple substitutions.

Supplementary Table 22. Test prediction performance on MdrDB_CoreSet (multiple substitutions). Mean performance (±std) over 5 repetitions are reported. The best is highlighted in **bold**.

Methods	RMSE	Pearson	AUPRC
**MLP**	2.202 ± 0.476	0.158 ± 0.111	0.18 ± 0.076

**DecisionTree**	1.548 ± 0.128	0.356 ± 0.067	0.412 ± 0.132
**ElasticNet**	1.347 ± 0.07	0.223 ± 0.029	0.22 ± 0.072
**Lasso**	1.331 ± 0.076	0.217 ± 0.04	0.217 ± 0.087
**SVR**	1.329 ± 0.101	0.159 ± 0.072	0.265 ± 0.144
**AdaBoost**	1.198 ± 0.087	0.457 ± 0.074	0.497 ± 0.042
**GradientBoost**	1.17 ± 0.091	0.506 ± 0.071	0.515 ± 0.045
**Bagging**	1.14 ± 0.08	0.538 ± 0.064	0.55 ± 0.079
**RandomForest**	1.132 ± 0.085	0.55 ± 0.069	0.566 ± 0.069
**ExtraTrees**	**1.057 ± 0.073**	**0.62 ± 0.068**	**0.637 ± 0.075**

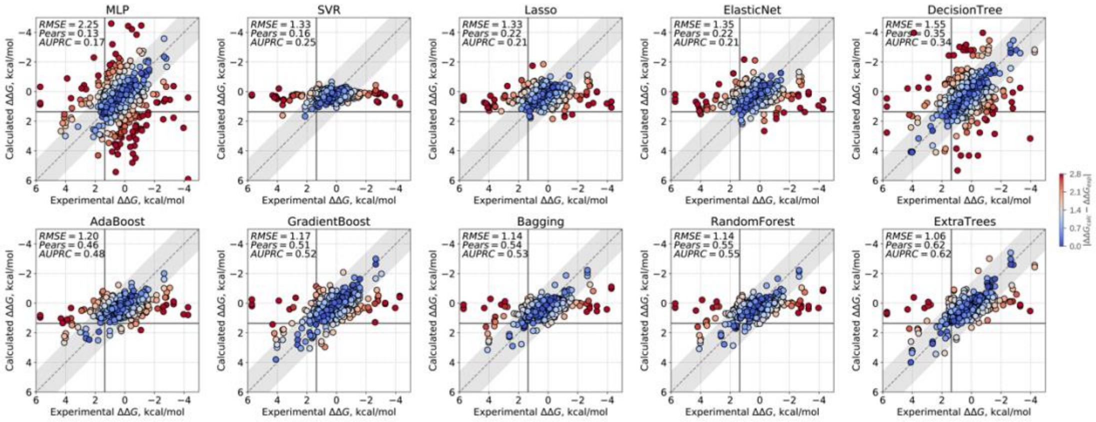

Supplementary Figure 22. Scatter plots of the experimental versus calculated ΔΔG values in Scenario 4.1. Each ΔΔG estimate is color-coded according to its absolute error w.r.t. the experimental ΔΔG value; at 300 K, the 1.4 kcal mol^{-1} error corresponds to a 10-fold error in the Kd change and 2.8 kcal mol^{-1} error corresponds to a 100-fold error in the Kd change.

Scenario 4.2: Training on single substitution mutations, fine-tuning on (deletion+indel+insertion+complex) mutations (fine-tune : test = 8 : 2).

In scenario 4.2, the machine learning methods are trained on the MdrDB_CoreSet (single substitution), then the model parameters are fine-tuned on approximately 80% of the samples in MdrDB_CoreSet (complex mutations, i.e., deletion, indel, insertion,

and complex), and then tested on the remaining complex mutations. Supplementary Table 23 shows the average prediction performance of all the competing machine learning methods over 5 repetitions, and the scatter plots of the experimental versus calculated ΔΔG values in one experiment are displayed in Supplementary Figure 23. Compared with the results of Scenario 3.2, the model uses a part of the complex mutation samples for fine-tuning, and the prediction performance is improved to a certain extent.

Supplementary Table 23. Test prediction performance on MdrDB_CoreSet (deletion+indel+insertion+complex). Mean performance (±std) over 5 repetitions are reported. The best is highlighted in **bold**.

Methods	RMSE	Pearson
**MLP**	4.203 ± 1.244	-0.006 ± 0.497
**DecisionTree**	1.627 ± 0.437	0.365 ± 0.173
**ElasticNet**	1.577 ± 0.164	0.473 ± 0.227
**Lasso**	1.449 ± 0.158	0.476 ± 0.244
**ExtraTrees**	1.363 ± 0.448	0.254 ± 0.319
**SVR**	1.357 ± 0.437	-0.005 ± 0.241
**Bagging**	1.227 ± 0.402	0.376 ± 0.248
**RandomForest**	1.206 ± 0.404	0.404 ± 0.252
**GradientBoost**	1.172 ± 0.438	0.477 ± 0.276
**AdaBoost**	**1.126 ± 0.474**	**0.54 ± 0.319**

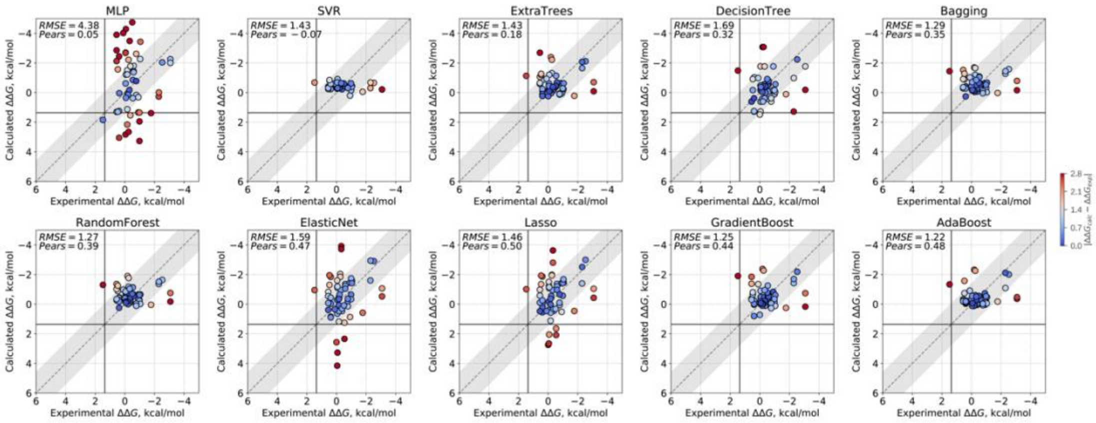

Supplementary Figure 23. Scatter plots of the experimental versus calculated ΔΔG values in Scenario 4.2. Each ΔΔG estimate is color-coded according to its absolute error w.r.t. the experimental ΔΔG value; at 300 K, the 1.4 kcal mol^{-1} error corresponds to a 10-fold error in the Kd change and 2.8 kcal mol^{-1} error corresponds to a 100-fold error in the Kd change.

Supplementary Note2: Web design and interface

Browse.

Users can browse all the curated and processed data through the Search MdrDB page, accessible via a quick-access button in the navigation bar. Data are presented in tabular form, with the following basic data given for each sample: MdrDB sample ID, UniProt ID, PDB ID, mutation type, mutation string, drug name, drug SMILES, $\Delta\Delta G$ value, and sample source. From this page, users can easily search, sort, filter, and download samples (Supplementary Figure 24A).

Search.

A full guide to the basic search and advanced search provided by MdrDB is contained in the tutorial available on the website. Here we summarize the key features.

With a basic search, users can query any of the following keywords directly in the search bar (Supplementary Figure 24A.❶):

- MdrDB ID: e.g., "MdrDB00158"
- UnitProt ID: e.g., "P00533"
- Mutation String: e.g., "G791S"
- Drug Name: e.g., "Embelin" (alternative names of drugs recorded in PubChem may also be used)

Advanced search (Supplementary Figure 24A.❷) is performed by prepending queries to keywords with an appropriate prefix, e.g., "MS:" corresponds to the MUTATION_SOURCE keyword. An example of a valid query is "MS:alphafold2".

The "*" wildcard symbol can be used to search in place of unspecified characters or values, e.g., "P:*G9*" or "T:*substitution".

Multiple (space-separated) keywords can be queried simultaneously with both basic and advanced searches. For example, UniProt ID and mutation string keywords can be searched simultaneously, with a query such as "P98170 R443C".

Display.

On the sample display page, users can view detailed sample information (Supplementary Figure 24A.❻-❾): (i) basic information, (ii) drug structure, (iii) biochemical features, and (iv) structure of wild type and mutant proteins. The basic information block (Supplementary Figure 24B), in addition to containing the information displayed in the browse and search page, also gives hyperlinks to the corresponding UniProt (UniProt ID), RCSB PDB (PDB ID) and PubChem (CID) entries. The drug structure block (Supplementary Figure 24A.❼) displays the two-dimensional (2D) structure of the drug. The biochemical features block (Supplementary

Figure 24A.❽) contains six types of features: ligand property features, protein property features, mutation environment features, protein-ligand interaction features, Vina features, and SASA features. The structure of wild type and mutation block (Supplementary Figure 24D.❷) displays the sequence around the mutation sites, overall structures of wild type and mutant proteins, and close-up views of the mutated sites. Color coding is used to indicate mutation site residues in the wild type (magenta) and mutant (orange) proteins. The two residues that are next to the mutation site are colored grey. The close-up view gives a clear picture of the changes in residues near the mutation site before and after the mutation. Users can download structure and feature files for each sample by clicking the download button on the upper right of the sample display page.

Download of data, figures, and tables.

All plots displayed on the website (e.g., pie charts, bar plots, and heatmap) can be downloaded in a variety of formats (JPG, PNG, and PDF) by clicking the button at the top right of the plot. On the download page of the MdrDB website, users can download metadata, structure files, processed biochemical features, and annotation files. MdrDB provides two dataset types to users for downloading: MdrDB_CoreSet and MdrDB_FullSet. MdrDB_CoreSet provides non-repetitive "UniProt-mutation-drug" samples whose features are averaged over all corresponding PDB features. MdrDB_FullSet provides all "UniProt-mutation-drug" samples, whose features are calculated based on each PDB structure.

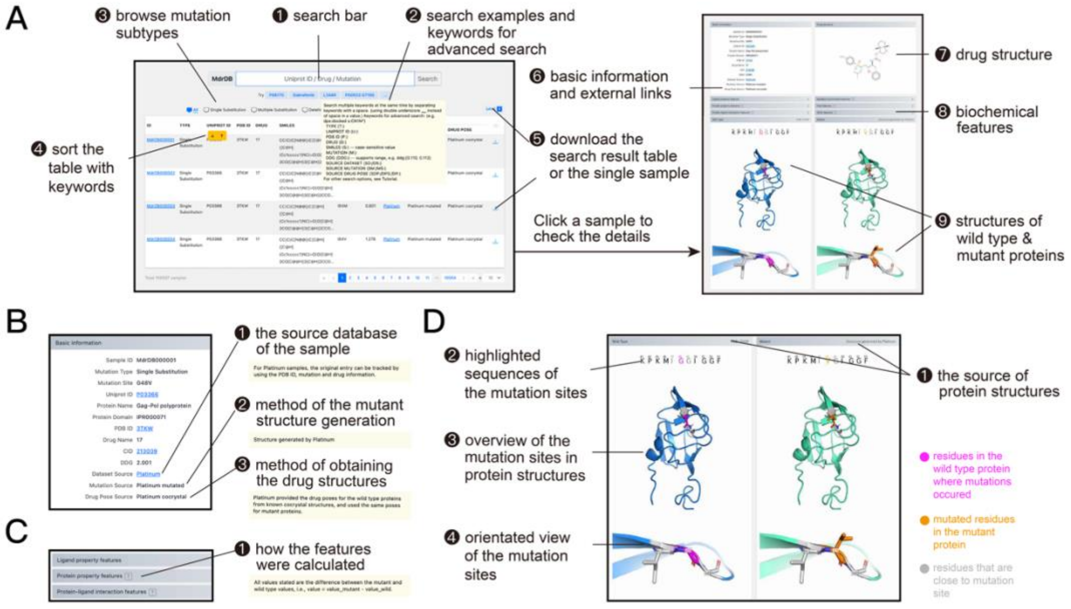

Supplementary Figure 24. The search and browse page and the sample display page of the MdrDB website. (A) Basic functions of the search and browse page and information are included in the sample display page. (B) Details of data tracks are in the "Basic Information" section on the sample display page. (C) Details of feature

calculation are in the “Features” section on the sample display page. (D) Details of different views of wild type and mutant structures are in the “Protein Structures” section on the sample display page.

Supplementary References

[1] Vitkup, D., Sander, C., & Church, G. M. (2003). The amino-acid mutational spectrum of human genetic disease. Genome biology, 4, 1-10.
